# Disability and ageing in China and India – decomposing the effects of gender and residence. Results from the WHO study on global AGEing and adult health (SAGE)

**DOI:** 10.1186/s12877-017-0589-y

**Published:** 2017-08-31

**Authors:** Jennifer Stewart Williams, Fredrik Norström, Nawi Ng

**Affiliations:** 1 Unit of Epidemiology and Global Health, Department of Public Health and Clinical Medicine, Faculty of Medicine, Umeå University, SE-901 87 Umeå, Sweden; 2 Research Centre for Generational Health and Ageing, University of Newcastle, Newcastle, Australia; 3 Centre for Demographic and Ageing Research, Umeå University, SE-901 87 Umeå, Sweden

**Keywords:** Inequalities, Residence, Rural, Developing countries, Oaxaca-Blinder, Decomposition

## Abstract

**Background:**

China and India are the world’s two most populous countries. Although their populations are growing in number and life expectancies are extending they have different trajectories of economic growth, epidemiological transition and social change. Cross-country comparisons can allow national and global insights and provide evidence for policy and decision-making. The aim of this study is to measure and compare disability in men and women, and in urban and rural dwellers in China and India, and assess the extent to which social and other factors contribute to the inequalities.

**Methods:**

National samples of adults aged 50 to 79 years in China (*n* = 11,694) and India (*n* = 6187) from the World Health Organization (WHO) longitudinal Study on global AGEing and adult health (SAGE) Wave 1 were analysed. Stratified multiple linear regressions were undertaken to assess disability differences by sex and residence, controlling for other biological and socioeconomic determinants of disability. Oaxaca-Blinder decomposition partitioned the two-group inequalities into explained and unexplained components.

**Results:**

In both countries women and rural residents reported more disability. In India, the gender inequality is attributed to the distribution of the determinants (employment, education and chronic conditions) but in China about half the inequality is attributed to the same. In India, more than half of the urban rural inequality is attributed to the distribution of the determinants (education, household wealth) compared with under 20% in China.

**Conclusions:**

Education and employment were important drivers of these measured inequalities. Overall inequalities in disability among older adults in China and India were shaped by gender and residence, suggesting the need for policies that target women and rural residents. There is a need for further research, using both qualitative and quantitative methods, to question and challenge entrenched practices and institutions and grasp the implications of global economic and social changes that are impacting on population health and ageing in China and India.

**Electronic supplementary material:**

The online version of this article (doi:10.1186/s12877-017-0589-y) contains supplementary material, which is available to authorized users.

## Background

China and India have the world’s largest country populations - estimated at 1.4 and 1.3 billion respectively. Together they are home to 31% of the global population. More than one third of adults aged 60 and above live in either China or India, and by 2050 this proportion will exceed 38% (three-quarters of 1 billion) [1–4]. Over the next one and half decades, about 65% of the Chinese health burden and 44% of the Indian health burden will be borne by adults aged 60 and above [5, 6]. These two population superpowers have similarly prominent global positions in the Asia-Pacific region, yet different demographic and epidemiological trajectories. Understanding socioeconomic disparities in health is particularly important for policy and planning and China and India are attracting increasing global attention in relation to the impact of social and economic factors on health [7]. Cross-country comparisons can allow global and national insights and provide evidence for decision-making. In this paper we analyse and compare inequalities in disability in older adults in China and India.

China initiated major industrial sector reforms in the early 1980s and India’s economic policy reforms began a decade later resulting in slower but nonetheless steady economic development. China’s late marriage and one-child policies have led to declines in fertility and changes in the population structure. Between 2000 and 2010, the ratio of the working-age (15–64 years) to non-working-age population in China rose from 2.2 to 3.1 while in India, the ratio increased from 1.5 to 1.75 in the same period. China is facing population ageing much earlier than India [7, 8].

Both countries are experiencing altered morbidity burdens due to increased life expectancies. China has made good progress with regard to successful infectious disease control, but now faces a rising epidemic of non-communicable diseases (NCDs). India is experiencing a double burden of both communicable and NCDs [9]. In China the largest share of the disease burden is due to NCDs [10].

Although China and India are similar in that they are both undergoing rapid social and economic transitions – including rising incomes, increasing rural to urban migration, changing roles for women in education and employment, and shifts from traditional extended family structures to smaller nuclear family units - there are notable differences. For example, primary education is almost universal in China but India is lagging in this regard. Between 1990 and 2005 the total share of the population attaining post-secondary education in China rose from 2% (2.7% male, 1.3% female) to 6.9% (7.8% male, 6.1% female). In contrast, the population of India is poorer and less well educated [11]. By most social and economic measures, the status of daughters, girls, and women is worse in India than China [7, 11, 12]. In China, labour force participation for women aged 15–64 years is higher than the average for middle-income countries (76% compared with 63%). Although younger women in India are becoming more educated, many older women are steeped in traditional customs and have less opportunities for educational advancement and professional employment than their male counterparts [8, 11]. Urbanisation is proceeding faster in China than in India and the sustainability of Chinese family agriculture is further challenged by the pressures of globalisation and climate change. China’s economic development has fuelled large-scale migration from rural to urban areas; younger workers are shifting from agriculture into industry and services in the cities and towns. Between 1990 and 2005, the percentage of people aged 60 and above working in agriculture in China increased from 5 to 10%. Although nationally China has reaped the economic benefits of increased productivity and mechanisation, the rural elderly are relatively poorer and more vulnerable than the urban elderly [6, 7, 13–17]. In India agriculture remains a major source of informal and low paid formal employment for older workers, particularly women, and poverty is high in rural areas [8, 11, 16, 18].

In 2006, the estimated shares of the labour force covered by mandatory pension schemes were 21% in China and 9% in India. In China approximately 68% of older people in cities and 21% in towns rely on pensions as a main source of support but India has no comparable social security [9]. Labour force participation among those aged 60 years and above is almost 50% in rural areas in India and this is mostly for low paid informal employment [8, 16, 19, 20]. As in many other traditional societies, extended family and kinship networks provide support for the elderly in China and India, particularly in rural areas. Changes in family and intergenerational relationships and women’s roles impact on the health and welfare of older people [21]. China is expanding coverage of government-funded health insurance [22] but in India healthcare is dominated by the private sector and affordability is a major barrier to access [9]. Only 15% of the population in India has access to formal health insurance, and 75% percent of all healthcare expenses are paid for directly by individuals, compared with 45% in China [8].

Older adults experience disability, or disablement, through decrements in physical, cognitive and social function. Disability coexists with many types of conditions, e.g. NCDs, infectious diseases, or events such as falls and stroke [23]. Under the International Classification of Functioning, Disability, and Health (ICF) framework ‘disability’ is understood to mean functioning according to body capacities that are modified by the individual’s physical and social environment and/or personal attributes [24–27]. This study uses the universally agreed ICF disability definition endorsed by the World Health Organization (WHO) [5, 27–29].

A few recent epidemiological studies have specifically compared and commentated on health and ageing in China and India using comparable self-reported data on health status (variously defined), risk factors, and chronic disease. In one of the first such studies the authors reported declines in health with increasing age, with a steeper gradient in India than in China [5]. Subsequent cross-country analyses of the health of adults aged 50 and above showed that respondents in China reported better health and less disability than those in India [6, 30]. The authors of a study of rural only adults in China and India identified significant wealth inequalities for functional limitation and disability (measured by activities of daily living) in both countries as well as better health status in rural China [14]. Urban rural differences were also highlighted in a more recent analysis that investigated the impact of out-of-pocket-health-expenditure on impoverishment in China and India. The authors concluded that, in both countries, residing in a rural area increased the likelihood of falling below the poverty line [31].

A huge body of literature refers to the now well-known social, economic and demographic factors, or social determinants, that create inequalities and inequities in health outcomes between people in all parts of the world. China and India are no exception [32, 33]. Individuals with higher education and income or wealth generally experience better health, and people who live in rural areas often have less proximity to healthcare and other services compared with their urban counterparts [34]. In many settings women and older adults are disadvantaged in terms of their health and social and economic circumstances, and people in rural areas face geographical and other barriers in accessing healthcare [27, 35–38].

In this study disability specifically refers to decrements in the individual’s body functions, or capacities to undertake activities that may or may not be linked to their underlying health condition(s) [39]. The aim is to measure and compare differences in disability in men and women, and in urban and rural dwellers, in adults aged 50 and above in China and India, and assess the extent to which social factors contribute to the inequalities. The objectives are to: 1) describe how gender, place of residence and other factors are associated with a standardised disability score, and 2) decompose the association between social determinants and inequalities in mean disability between men and women, and between urban and rural residents. The country comparison is timely because health and disability may well become significant economic impediments for China and India [7, 40]. The decomposition method is important because it shows how social and other determinants contribute to the measured inequalities.

## Methods

### Data collection

The data source is the WHO longitudinal Study on global AGEing and adult health (SAGE) Wave 1 (2007-2010). WHO-SAGE Wave 1 employed a multistage stratified random sampling strategy whereby stratification was based on the size of the first unit of selection - provinces in China and states in India. The probability proportional to size sampling method was used to select primary sampling units - towns in China and villages in India - and households were selected randomly within these units. All adults aged 50 and over in selected households completed the individual questionnaire which is the data source for this study. Post-stratification weights were used to adjust for age and gender distributions and non-response [41]. WHO-SAGE data sets are in the public domain. Details of WHO-SAGE are given elsewhere [42].

### Study variables

The study used a rigorous WHO measure of disability taken from questions about health and functioning. The questions covered eight domains of health-related function: vision; mobility; self-care; cognition; interpersonal activities; pain and discomfort; sleep and energy, and affect [43]. Respondents rated the amount of difficulty they had experienced in each domain in the previous 30 days using the categories: no difficulty, mild difficulty, moderate difficulty, severe difficulty and extreme difficulty. See Additional file 1: Appendix B.

Item Response Theory (IRT) was used to develop a calibrated disability score (the dependent variable) which was derived using a partial credit model with item calibration. The same method has been used in a number of other studies [5, 27, 28, 39]. (See Additional file 2: Appendix A). Chi-squared statistics were calculated to determine how well each item contributed to the overall disability measure. Raw scores were transformed into a continuous cardinal scale with a range of 0 (functionally able) to 100 (highest disability) [44].

The literature on the social determinants of health and disability informed the selection of independent variables [25, 27, 36]. With the exception of age, the independent variables were all categorical. The relationship between age and disability was linear and age is expressed continuously in single year increments. Other independent variables are: gender (men vs. women); marital status (never married/cohabiting vs. separated/divorced/widowed/single); employment (currently working vs. not currently working vs. never worked); education level (no primary schooling vs. completed primary school vs. completed secondary-high school vs. completed university/college); place of residence (urban vs. rural) and household wealth quintiles. A hierarchical ordered probit model [45] was used to develop a relative index of household wealth from which country-specific wealth quintiles were statistically generated in the raw data based on household asset ownership of durable goods, dwelling characteristics and access to services such as improved water, sanitation, and cooking fuel. Wealth quintiles provide an alternative measure of income and assets that is less likely to be biased by contextual differences than measures based on self-reported income [45]. Although in some studies quintile 1 denotes the least wealthy, here quintile 1 represents the wealthiest group and quintile 5 the least wealthy group.

Given that NCDs are contributors to disability in older adults we expected confounding because of association between NCDs and social factors [46, 47]. A ‘chronic conditions’ variable that measured up to five possible self-reported NCDs - depression, arthritis, angina, asthma and diabetes – is included. This is expressed as: zero vs. one vs. two or more chronic conditions.

### Study sample

The available samples from individual SAGE Wave 1 respondents were 15,050 in China and 12,198 in India. First only respondents aged 50 years and above were selected in line with the WHO distinction between younger and older people in developing countries [42]. The final country study samples comprised 11,694 respondents in China (5488 men and 6206 women) and 6187 respondents in India (3111 men and 3076 women). Exclusion criteria were: unfinished interviews (237 in China and 968 in India); age under 50 years (1636 in China and 4670 in India) and missing data on study variables (705 in China and 45 in India). At least 99% of records in both countries had complete data; there was no evidence of bias due to missing data. After checking for heteroscedasticity in the residuals, records of respondents aged 80 years and above were excluded at the next stage (778 in China and 328 in India). This is explained further in the Statistical analysis section.

### Ethics statement

WHO-SAGE study was approved by the Ethics Review Committee, World Health Organization, Geneva, Switzerland; the Ethics Committee, Shanghai Municipal Centre for Disease Control and Prevention, Shanghai, China, and the Institutional Review Board, International Institute of Population Sciences, Mumbai, India. Approval covered all procedures undertaken as part of the study. Written informed consent was given by all individual participants.

### Statistical analysis

The samples of men and women in China and India are described by socio-demographic characteristics and disability. Actual numbers of observations and weighted proportions (for categorical variables) are given. Tests of significance (Chi-squared for categorical variables and t-tests for continuous variables) are used to compare the characteristics of women and men in each country. The *p*-values are reported in the tables.

Univariable analysis was undertaken to select independent variables for the multiple linear regression with the inclusion criterion set at *p* < 0.05. No candidate variables were removed. Model assumptions for linear regression were checked. The only violation was heteroscedasticity in the residuals in records where age > = 80. For this reason, the age range for all analyses was 50 to 79 years inclusive.

Pairs of categorical variables were assessed for possible interactions i.e. to see whether the effect of one independent variable differed according to different levels of another independent. Interaction between gender and residence was observed (*p* < 0.05); the models were stratified by gender and residence to further investigate these effects. Multicollinearity was tested using the variance inflation factor (VIF) statistic (given in tables). Results of VIF < 5 indicate reasonable independence among predictor variables.

Stratification provided the basis for Oaxaca-Blinder decomposition [48] based on ordinary least squares regression. The method partitions the difference between two group means into a part that arises because the groups have different characteristics, and a part that is due to the differential effects of the characteristics in the groups. The first part is called the ‘explained’ component and the second the ‘unexplained’ component. The method is explained in greater detail elsewhere [49, 50]. See Additional file 3: Appendix C.

Four two-group decompositions were undertaken: inequality in mean disability scores between men and women in China; inequality in mean disability scores between men and women in India; inequality in mean disability scores between urban and rural residents in China, and inequality in mean disability scores between urban and rural residents in India. The decomposition methodology involves constructing counterfactual equations. For the gender inequality, for example, the decomposition constructs an equation that substitutes the intercept and coefficients from the men’s regression into the women’s regression. This approach allows us to distinguish between the part of the inequality attributed to the determinants in the model (i.e. the explained part) from the remaining part attributed to the effects of the coefficients (i.e. the unexplained part). The *p*-values show statistical significance.

Contributions (positive and negative) made by each of the determinants to the measured two-group inequality are reported. Positive contributions support the direction of the inequality and negative contributions offset the difference or gap in the disability meaurements.

All analyses reported here include SAGE country weights made available by WHO. STATA Version 13 (StataCorp, 2013) was used for all statistical analyses.

## Results

There were statistically significant differences in the frequencies between men and women for marital status (*p* < 0.01), education (*p* < 0.01), employment (*p* < 0.01), residence (*p* < 0.05) and chronic conditions (*p* < 0.01) in both China and India, and age in India (*p* < 0.01) (Table 1). Household wealth status was not significantly different between men and women in either country. The disability score was higher in India (men: 42.5 and women: 49.4) than in China (men: 29.2 and women: 33.2) and significantly higher in women than in men for both countries (*p* < 0.01).

**Table 1:** Characteristics of the study population, adults aged 50-79 years, China and India, Sage Wave 1, 2007-2010

	China (*n* = 11,694)		India (*n* = 6187)	
	Men (*N* = 5488)	Women (*N* = 6206)	Men (*N* = 3111)	Women (*N* = 3076)
	N (^a^ %)	N (^a^ %)	N (^a^ %)	N (^a^ %)
Marital status				
Married/Cohabiting	4985 (91.6)***	5008 (82.5)	2758 (92.2)***	1941 (64.9)
Separated/divorced/widowed/single	503 (8.4)	1198 (17.5)	353 (7.8)	1135 (35.1)
Education				
Completed college/university	353 (6.1)***	176 (2.9)	258 (8.9)***	61 (1.6)
Completed secondary	2157 (38.8)	1827 (28.3)	896 (30.9)	268 (7.1)
Completed primary	1299 (25.0)	1074 (18.3)	553 (18.3)	342 (11.5)
No primary	1679 (30.2)	3129 (50.5)	1404 (41.9)	2405 (79.9)
Employment				
Currently working	2700 (54.8)***	2033 (35.0)	1924 (66.6)***	674 (20.6)
Not currently working	2334 (40.2)	3203 (52.9)	1068 (30.8)	797 (26.5)
Never worked	454 (5.0)	970 (12.2)	119 (2.6)	1605 (51.9)
Household wealth status				
Wealthiest quintile	1081 (22.2)	1254 (22.5)	779 (24.3)	766 (23.3)
Second wealthiest quintile	1169 (24.1)	1274 (23.6)	698 (21.1)	643 (18.3)
Mid quintile	1122 (20.4)	1228 (20.5)	559 (18.5)	580 (19.4)
Second poorest quintile	1108 (18.2)	1208 (17.7)	574 (19.0)	583 (20.1)
Poorest quintile	1008 (15.1)	1242 (15.8)	501 (17.1)	504 (18.9)
Residence				
Urban	2512 (43.7)***	3209 (51.5)	757 (28.8)**	844 (29.7)
Rural	2976 (56.3)	2997 (48.6)	2354 (71.2)	2232 (70.3)
Chronic conditions				
No conditions	3900 (71.2)***	3852 (61.2)	1482 (47.8)***	1231 (39.5)
One condition	1241 (23.2)	1761 (29.2)	904 (26.7)	992 (32.4)
Two or more conditions	347 (5.5)	593 (9.6)	725 (25.5)	853 (28.2)
	Mean (SD)	Mean (SD)	Mean (SD)	Mean (SD)
Age mean in years (SD)	62.0 (8.1)	61.7 (8.2)	61.0 (7.7)***	60.1 (7.4)
Disability score mean (SD)	29.2 (15.5)***	33.2 (14.9)	42.5 (15.1)***	49.4 (12.9)

ƛ^2^ tests for categorical variables and two-sample, two-sided t-tests for continuous variables with **p-value < =0.05; ***p-value < =0.01

^a^ survey sampling weights used to give percentage estimates. Percentages may not sum due to rounding. *SD* standard deviation

For the analysis of disability, the full (un-stratified) models show that in both countries, women and rural residents who were older and poorer, had incomplete primary education, were not employed, and had multiple chronic conditions, reported more disability and that these differences were statistically significant. Martial status was not significant in either country. The stratified linear regressions reveal specific associations with disability separately for men and women and for urban and rural residents.

In the gender stratification, older age was statistically significant and positively associated with disability for men and women in both China (Table 2) and India (Table 3). Compared with being married or cohabiting, being separated, divorced, widowed or single, was statistically significant in association with disability for men only in China and for women only in India. In both countries having had no primary education, compared with having completed college or university, was statistically significant in association with disability. Having never worked, compared with not currently working, was statistically significant in association with disability in men and women in both countries; in India the association was significantly higher for men. Being in households with greater wealth was also statistically significant and protective of disability in men and women in both China and India. Rural residence was statistically significant and similarly associated with disability for men and women in China, but the association was statistically significant only for men in India. Having multiple diagnosed chronic conditions was statistically significant in association with disability for men and women in both China and India.

**Table 2:** Multiple linear regression of factors associated with disability, full and stratified models, adults aged 50-79 years, China, SAGE Wave 1

	Full model	Model stratified by gender		Model stratified by residence	
	Total (*N* = 11,694)	Men (*N* = 5488)	Women (*N* = 6206)	Urban (*N* = 5721)	Rural (*N* = 5973)
	Coefficient (95% CI)	Coefficient (95% CI)	Coefficient (95% CI)	Coefficient (95% CI)	Coefficient (95% CI)
Intercept	−9.13 (−12.9,5.34)	−9.98 (−14.5,-5.42)	−6.04 (−10.1,-1.95)	−8.24 (−12.8,-3.64)	−12.3 (−19.2,-5.42)
Age	0.38 (0.33,0.43)	0.38 (0.31,0.45)	0.38 (0.34,0.43)	0.38 (0.30,0.45)	0.39 (0.33,0.46)
Gender (Reference men)					
Women	2.35 (1.73,2.97)	NA	NA	1.52 (0.64,2.40)	3.12 (2.19,4.04)
Marital status (Reference married/cohabiting)					
Separated/divorced/widowed/single	0.97 (−0.13,2.06)	2.50 (0.54,4.47)	0.39 (−0.49,1.27)	0.91 (0.02,1.80)	1.00 (−1.07,3.07)
Education (Reference college/university)					
Completed secondary	2.71 (0.87,4.55)	2.68 (0.16,5.21)	3.06 (1.09,5.03)	2.20 (0.40,4.01)	10.3 (5.60,15.0)
Completed primary	4.22 (1.91,6.52)	3.74 (0.85,6.62)	5.27 (2.91,7.64)	4.91 (2.22,7.60)	10.2 (5.74,14.7)
No primary	4.87 (2.71,7.04)	4.65 (1.87,7.43)	5.49 (3.26,7.72)	5.09 (2.64,7.54)	11.4 (6.77,16.0)
Employment (Reference currently working)					
Not currently working	3.28 (2.41,4.14)	4.26 (2.89,5.64)	2.07 (0.97,3.16)	3.26 (1.81,4.71)	3.47 (2.19,4.74)
Never worked	3.40 (1.95,4.84)	3.74 (1.80,5.68)	2.75 (1.23,4.27)	3.51 (0.45,6.56)	3.41 (1.73,5.10)
Household wealth (Reference wealthiest)					
Second wealthiest quintile	4.64 (2.55,6.74)	5.23 (2.95,7.50)	4.06 (1.67,6.45)	3.54 (0.82,6.26)	6.34 (3.61,9.08)
Mid quintile	5.98 (3.86,8.10)	6.88 (4.69,9.08)	5.06 (2.66,7.45)	5.20 (2.39,8.01)	7.15 (4.38,9.92)
Second poorest quintile	6.27 (4.17,8.37)	6.99 (4.92,9.06)	5.51 (3.02,8.00)	7.92 (5.06,10.8)	6.28 (3.55,9.02)
Poorest quintile	7.89 (5.37,10.4)	8.98 (6.40,11.6)	6.76 (4.01,9.51)	8.54 (5.74,11.4)	8.37 (4.64,12.1)
Residence (Reference urban)					
Rural	5.38 (4.11,6.64)	5.02 (3.46,6.59))	5.54 (4.26,6.82)	NA	NA
Chronic conditions (Reference no conditions)					
One condition	7.21 (6.63,7.79)	8.00 (7.07,8.93)	6.48 (5.68,7.28)	6.84 (6.05,7.64)	7.44 (6.56,8.32)
Two or more conditions	12.8 (11.7,13.9)	12.6 (11.0,14.2)	12.7 (11.6,13.9)	13.7 (12.2,15.2)	11.2 (9.62,12.8)
R-squared	0.28	0.26	0.27	0.29	0.23
Variance Inflation Factor (VIF)	1.38	1.35	1.36	1.40	1.30

Note: Survey sampling weights applied

*NA* not applicable

**Table 3:** Multiple linear regression of factors associated with disability, full and stratified models, adults aged 50-79 years, India, SAGE Wave 1

	Full model	Model stratified by gender		Model stratified by residence	
	Total (*N* = 6187)	Men (*N* = 3111)	Women (*N* = 3076)	Urban (*N* = 1601)	Rural (*N* = 4586)
	Coefficient (95% CI)	Coefficient (95% CI)	Coefficient (95% CI)	Coefficient (95% CI)	Coefficient (95% CI)
Intercept	10.4 (5.56,15.1)	9.20 (1.80,16.6)	15.3 (9.84,20.8)	7.37 (−2.23,17.0)	14.4 (9.55,19.3)
Age	0.29 (0.22,0.35)	0.30 (0.19,0.41)	0.24 (0.16,0.32)	0.33 (0.17,0.47)	0.28 (0.22,0.34)
Gender (Reference men)					
Women	2.85 (1.61,4.10)	NA	NA	4.63 (1.38,7.87)	2.45 (1.15,3.74)
Marital status (Reference married/cohabiting)					
Separated/divorced/widowed/single	1.15 (−0.30,2.60)	−1.23 (−3.66,1.21)	2.10 (0.35,3.85)	1.00 (−2.99,5.19)	1.03 (−0.32,2.37)
Education (Reference college/university)					
Completed secondary	2.63 (0.21,5.05)	1.95 (−0.89,4.80)	4.66 (0.75,8.57)	2.93 (−1.08,6.94)	1.91 (−1.36,5.18)
Completed primary	3.35 (0.05,6.66)	2.32 (−1.57,6.22)	6.49 (2.09,10.9)	2.77 (−3.88,9.43)	3.13 (−0.14,6.41)
No primary	5.04 (2.30,7.78)	4.40 (1.17,7.64)	7.45 (3.50,11.4)	4.36 (0.89,9.60)	4.67 (1.54,7.80)
Employment (Reference currently working)					
Not currently working	4.23 (2.92,5.33)	5.09 (3.13,7.04)	2.67 (0.94,4.41)	2.61 (−0.26,5.48)	4.99 (3.90,6.09)
Never worked	4.38 (2.65,6.11)	10.9 (7.53,14.3)	2.88 (1.20,4.57)	2.49 (−1.67,6.65)	4.84 (3.15,6.53)
Household wealth (Reference wealthiest)					
Second wealthiest quintile	2.18 (0.52,3.84)	3.28 (0.79,5.77)	0.85 (−0.81,2.51)	4.23 (0.58,7.87)	1.16 (−0.25,2.58)
Mid quintile	3.24 (1.32,5.15)	3.46 (1.20,5.73)	2.89 (0.40,5.38)	3.72 (−0.49,7.94)	2.99 (1.12,4.86)
Second poorest quintile	3.59 (1.75,5.42)	3.80 (1.32,6.28)	3.19 (0.92,5.46)	1.38 (−3.36,6.11)	3.88 (2.29,5.46)
Poorest quintile	5.92 (4.11,7.72)	5.25 (2.62,7.88)	6.17 (4.34,7.99)	8.67 (4.32,13.0)	5.14 (3.21,7.07)
Residence (Reference urban)					
Rural	2.57 (0.31,4.83)	3.14 (0.70,5.59)	2.01 (−0.72,4.74)	NA	NA
Chronic conditions (Reference no conditions)					
One condition	7.28 (5.95,8.62)	7.55 (5.64,9.46)	7.05 (5.50,8.60)	7.53 (4.09,11.0)	7.02 (5.87,8.17)
Two or more conditions	12.8 (11.1,14.5)	13.3 (11.1,15.6)	12.3 (10.4,14.3)	14.8 (11.0,18.6)	11.9 (10.7,13.2)
R-squared	0.31	0.29	0.26	0.32	0.30
Variance Inflation Factor (VIF)	1.45	1.41	1.35	1.47	1.43

Note: Survey sampling weights applied

*NA* not applicable

In the residence stratification, older age was statistically significant and positively associated with disability for urban and rural residents in both countries. In rural China and urban India, male gender was statistically significant and protective of disability. Higher education was statistically significant and protective of disability for both urban and rural residents, particularly in rural China. Employment was statistically significant and protective of disability regardless of residence in China but statistically significant only for rural residents in India. Household wealth was statistically significant and protective of disability for both urban and rural residents in both countries. Having multiple diagnosed chronic conditions was statistically significant in association with disability in both countries, and particularly for urban residents.

Factors positively associated with disability in both women and men and urban and rural residents in China and India were older age, lower household wealth, and multiple diagnosed chronic conditions. In the gender stratification, rural residence was associated with disability. In the residence stratification, female gender was associated with disability, particularly in rural China and urban India. In China, being male and married was protective of disability and in India, being female and married was protective of disability. Current employment and higher education were protective of disability in both stratifications. However in India, higher education was particularly protective of disability in women, and employment was more strongly protective of disability in men and in China, higher education was more protective among rural residents.

The stratified linear regressions show associations between the determinants and disability in gender and residence sub-groups in China and India. In the post-regression decompositions inequality in disability i.e. either between men and women or urban and rural residents, is the outcome. Each regression-based decomposition shows the extent to which the determinants explain the measured inequality.

When reporting the results of the Oaxaca-Blinder decomposition it usual to highlight the findings to show how much of the measured inequality is ‘explained’ or not by the distribution of the determinants in each group. This summary information can communicate clear messages to policy-makers.

Table 4 shows the gender decompositions for China and India. On average in both countries, women had more disability. The inequality was greater in India than in China. In China, about half of the gender inequality can be explained by differences in the distribution of the determinants. In other words out of a difference of 3.96 in disability points, about 1.91 (disability points) were due to differences in the distribution of the determinants in the two groups of men and women. If the determinants had been equally distributed in China the disability inequality would have been 1.91 points lower. In India, the contributions by the individual determinants (5.91) exceeds the inequality (5.86). This means that if the distribution of the determinants were the same for women as it was for men, men would have had more disability than women. We would then expect that on average women’s disability would have been 0.05 points less than men’s.

**Table 4:** Decomposition of gender inequality in disability by China and India, adults aged 50-79 years, SAGE Wave 1

	China (*N* = 11,694)	India (*N* = 6187)
	Mean (CI)	Mean (CI)
Mean disability score women	33.2 (32.8,33.6)	49.0 (48.5,49.5)
Mean disability score men	29.2 (28.8,29.6)	43.1 (42.6,43.7)
Difference in mean disability	3.96 (3.40,4.51)	5.86 (5.16,6.56)
Difference that is explained by the determinants	1.91	5.91
Difference that is not explained by the determinants	2.05	−0.05
Explained Contribution	Contribution	Contribution
Determinants		
Age	−0.09	−0.33***
Marital status	0.22***	−0.22
Education	0.37***	1.38***
Employment	0.66***	4.37***
Household wealth	0.05	0.03
Residence	−0.17***	−0.06**
Chronic conditions	0.87***	0.7***
Total Explained Contribution	1.91***	5.91***

**p-value < 0.05 ***p-value < 0.01

*CI* 95% confidence interval

Age and residence were both negative contributors, which means that they offset or decreased the inequality. Women were younger and tended to live in urban areas and younger age and urban residence were protective of disability. Overall the gender inequalities in both China and India are mostly explained by the distribution of multiple chronic conditions, unemployment and lower education in women compared with men (*p* < 0.01).

Table 5 shows the decompositions by urban and rural residence for China and India. Overall the disability inequality disfavours rural residents, with the difference larger in India. In China about 20% of the residence inequality is explained by the distribution of the determinants compared with over half in India. Given a difference of 3.39 disability points between urban and rural residents in China, just 0.65 (disability points) were due to differences in the distribution of the determinants in the two residence groups. In India out of the difference of 4.12 disability points between urban and rural residents, 2.18 points were due to differences in the distribution of the determinants in the two residence groups. Therefore most of the residence inequality in India was due to the distribution of the determinants while a large part of the residence inequality in China was attributed to unobserved factors.

**Table 5:** Decomposition of residence inequality in disability by China and India, adults aged 50-79 years, SAGE Wave

	China (*N* = 11,694)	India (*N* = 6187)
	Mean (CI)	Mean (CI)
Mean disability score rural	33.0 (32.6,33.4)	47.1 (46.7,47.5)
Mean disability score urban	29.6 (29.2,30.0)	43.0 (42.3,43.7)
Difference in mean disability	3.39 (2.83,3.94)	4.12 (3.30,4.94)
Difference that is explained by the determinants	0.65	2.18
Difference that is not explained by the determinants	2.74	1.94
Explained Contribution	Contribution	Contribution
Determinants		
Age	−0.60***	−0.04
Gender	−0.09***	−0.05
Marital status	−0.01	−0.02
Education	1.26***	1.64***
Employment	−0.85***	−0.31**
Household wealth	1.89***	0.98**
Chronic conditions	−0.95***	−0.03
Total Explained Contribution	0.65**	2.18***

**p-value < 0.05 ***p-value < 0.01

*CI* 95% confidence interval

Education and household wealth were major contributors to the residence inequalities in both China and India, regardless of offsetting contributions by age, residence, marital status, employment, and chronic conditions. This means that although factors such as younger age, male gender, marital status, employment, and chronic conditions favoured rural residents, education and household wealth were dominating contributors to the inequality that disfavoured rural residents. As with the gender inequality, this was more evident in India than in China. Both the gender and residence decompositions therefore explain a large share of the disability inequalities in India. However, in China the inequalities are also attributed to ‘other’ factors not specified in our models. Further reference to this is given in the Discussion.

## Discussion

This study of adults aged 50 to 79 years in China and India shows how gender, residence, age, marital status, education, employment, household wealth and chronic conditions are associated with disability. The decomposition further elucidates how these determinants separately contribute to inequalities in disability between men and women and urban and rural residents.

There are several notable findings. Firstly, disability was higher in India than in China. Secondly, women reported more disability than men with the difference larger in India than China. Thirdly, rural residents reported more disability than urban residents, with the difference again larger in India. Fourthly, in India the gender inequality in disability was attributed to the determinants, predominantly employment, education and diagnosed chronic conditions. Only half of the inequality was attributed to the same determinants in China. Lastly, in India, just over half of the inequality in disability between rural and urban residents was attributed to the determinants, largely education and household wealth. However in China, less than 20% of the inequality in disability between rural and urban residents was attributed to the determinants.

The results point to greater inequalities in disability between men and women and urban and rural residents, in India than in China. The decomposition analysis shows that these inequalities are mainly attributed to social factors such as employment and education in India. It is well-known that women in India still face gender inequalities in education and employment [12]. Women in China fare relatively better in this regard, and gender equality is promoted within the national agenda [51]. Another reason for the difference is that Chinese culture has a strong Communist norm whereby there is an expectation that all working age men and women should be in the workforce contributing to national income [7, 11, 12].

### Disability differences between China and India

The finding of better population health in China compared with India is not new [5, 6, 10, 29, 30, 52]. What this study adds however is: firstly, a direct comparison between China and India using a standardised measure of disability that encapsulates broad multifaceted aspects of health and function in older adult populations, and secondly, informed explanation of the measured inequalities by country, gender and residence.

Economic development is one explanation for the disability difference between China and India. China’s per capita income exceeds that of India. China emerged as a dominant global economic force following a series of major market-orientated reforms that began in the 1980s. By contrast, India began a slower more gradual economic transformation in the 1990s. Between 1990 and 2005 China’s average per capita growth was 8.7% compared with India’s 4%. China’s economic growth was largely responsible for a decline in the poverty rate from 64% in the early 1980s to 10% in 2004. The bi-directional association between economic growth and population health is well-established. Improved health leads to economic growth and economic growth leads to improved health [7, 52, 53].

In China healthcare is underwritten by the government and recent reforms are aimed at increasing health insurance coverage. China’s success so far has been notable with healthcare now made more accessible and affordable across the population, and coverage extended to older adults [22]. However in India the majority of healthcare is delivered by a largely unregulated private sector. For large sections of the population in India, including older people and the poor, affordability is an impediment to healthcare access [9, 52].

### Disability differences between men and women in China and India

Differences in self-reported health measures between men and women have been documented in studies undertaken in many countries [37] including China and India [14]. Gender health inequality can vary by a decade. A study of China and India for example, showed that in both countries, even after correcting for possible reporting biases, the health scores for women aged 50-59 were similar to those for men aged 60–69, and for women aged 60-69 health scores were similar to men aged 70 and above [6]. The reasons for gender inequality in health are still unclear, and debate about the issues is ongoing. There is still much to learn about the pathways and mechanisms through which social determinants differently impact on the health of women and men.

A possible explanation put forward to explain women’s relatively poorer health concerns life expectancies. It has been argued that although women live longer than men they experience longer periods of poor health including non-life threatening chronic conditions such as arthritis and depression. Differences in biological factors and social determinants may be contributing to the measured health differences between women and men. The phenomenon, known as the “feminisation of ageing”, is now widely recognised and deserving of policy attention, particularly in large populous ageing countries such as China and India [6, 30, 37].

The decomposition shows that the distribution of education and employment in the country samples explains gender inequalities in both countries, but particularly in the Indian sample: 23.4% and 73.9% for education and employment compared with 19.4 and 34.6% respectively in China. The study sample characteristics show that in China about half of the female respondents (vs. 30% of men) had not completed primary school, and only 35% of women (vs. 55% men) were currently working. In India 80% women vs. 42% men had never completed primary school and only 21% women vs. 67% men were currently working. Lower attainment in education and employment among women in India is a factor contributing to women’s poorer health relative to men’s [9, 20, 54]. In India there are entrenched cultural, religious and ethnic norms that favour men over women in many areas of public and private life including education and employment [17]. While traditional gender roles in China have disadvantaged women in the past this is slowly changing as the country embraces globalisation and moves towards a more modern open society [4, 7].

The decomposition analysis also shows that, in both countries, the distribution of reported chronic conditions (or NCDs) in the country samples helps explain the disability gender inequalities in both countries. The notable contribution of chronic conditions to disability is expected because chronic conditions are a reason for decrements in the health in older people. However, it is interesting to note the relatively larger contribution by China compared with India (45.5% vs. 12.52%). Possible reasons for this country difference include the patterning of NCD risk factors, dissimilar stages of epidemiological transition and differences in healthcare coverage [4, 53].

The benefits of China’s economic growth need to be balanced against the high burden of NCDs [7, 10]. Non-communicable disease risk factor behaviours, such as tobacco use, harmful alcohol practices, poor diet and sedentary lifestyles are associated with major social changes such as increasing incomes, rising affluence urbanization, and industrialisation. These global trends started earlier and have been occurring more rapidly in China than in India [5, 53]. India’s health burden is still dominated by communicable, maternal, perinatal, and nutritional conditions accounting for 37% of all mortality, compared with just 7% in China [52]. China’s reforms in moving towards universal healthcare coverage have so far improved access to healthcare for older adults. Better access to diagnosis and treatment services for chronic conditions in China is a reasonable explanation for China’s relatively higher chronic conditions contribution to the measured inequality [22].

We show that education, employment and chronic conditions help explain gender inequalities in China and India but only about 50% of the gender inequality in China was explained by the determinants in the regressions. The results highlight important policy messages for India in regard to addressing women’s disadvantage with respect to chronic conditions, employment and education.

There are other reasons for China’s large ‘unexplained’ gender inequality. The gains from China’s economic growth and prosperity have not been equally distributed across population sub-groups. Social welfare benefits including age pensions and health insurance are not yet universal, and some older women and rural residents have limited access to affordable health care compared with other sections of the population [16, 22, 53].

There are also cultural reasons for gender inequalities. China has traditionally favoured the continuity of the male line. Despite increased gender inequity in education and employment, females are still considered inferior to males in many areas of Chinese society [11, 55]. Although this is slowly changing for younger women, the effects of cultural gender bias on the female psyche and as part of that, women’s health and well-being, must not be underestimated.

### Disability differences between urban and rural residents in China and India

In many parts of the world people who live in rural compared with urban areas have less access to health and other services and rural residents [35]. Higher disability in rural India compared with rural China reflect different rates and patterns of urbanisation [7]. According to the World Bank Data Bank in 2015, 56% of the population in China lived in urban areas compared with 33% in India (http://data.worldbank.org/indicator/SP.URB.TOTL.IN.ZS). The results of surveys conducted amongst older people in India’s towns and villages show a high prevalence of risky behaviours such as tobacco and alcohol use and inadequate physical activity. Healthcare in India is limited not only by limited coverage of health conditions but also by poor coverage to rural areas [6, 9, 20]. In contrast China’s rural health insurance program that began in 2003 has improved healthcare access for older rural residents [22].

The decomposition shows that the distribution of education and household wealth helps explain the disability residence inequalities in both countries, but notably more so in the China sample. People living in rural areas tend to be poorer and have less education than their counterparts in the cities [32, 33]. Growing income and education disparities between rural and urban residents have been observed with restrictions on mobility limiting further opportunities for people living in rural areas [53]. While the same overall trends are occurring in India, the factors that underpin rural to urban migration, and demographic and epidemiological transition across and within India’s many states are varied and complex. Urbanisation is constrained by geographic alignment with ethnic and religious boundaries across many rural areas. The majority of India’s older adults still reside in rural areas, many with some levels of family support.

It is interesting to note that the distribution of chronic conditions significantly offsets the residence inequality in China. This is possibly a reflection of China’s economic and healthcare reforms providing improved access to diagnosis and treatment in rural areas [7, 22].

Less than 20% of the urban rural inequality in China was explained by the determinants, compared with just under 50% in India. Factors not included in the model, that may have influenced this difference, include necessary regional infrastructure integral to the rollout of China’s economic and social reforms; urban areas often benefit from social welfare reforms before rural areas. The rural elderly in China traditionally rely more heavily on family support than the urban elderly and this is important for health and well-being. Yet rapid migration of young adults to the cities has caused disruption of family structures in rural areas of China [16, 22].

### Strengths of the study

This study is important for a number of reasons. Firstly, the data derive from information on health, well-being and behaviours collected from nationally representative cohorts of older adult residents in China and India using standardised questionnaire instruments. Second, this study uses a rigorously defined disability construct that captures health decrements experienced in everyday life, irrespective of aetiology. Disability may be due to NCDs such as CVD, diabetes or arthritis, as well as functional impairments such as loss of sight, hearing or mobility that impact on activities of daily living. Thirdly, the decomposition method shows the impact of social and demographic factors on health differences between men and women and residents of urban and rural areas. This allows us to disentangle the determinants of health inequalities between population sub-groups. Efforts to intervene on these determinants might bring policy-makers closer to closing the gaps in observed health inequalities.

### Limitations of the study

This analysis is cross-sectional and therefore cannot address the causality (in either direction) between the determinants and our measure of disability. The observed inequality in disability might reflect true differences in disability across the study population. Nevertheless, it might also be due to differential health reporting by men and women with different education levels. Our results showed that compared with India, a relatively high proportion of the overall inequality by gender and by residence in China was not explained by the determinants in the models. This suggests a need for further research to identify other factors that contribute to these inequalities. We acknowledge that there is evidence of under-reporting of chronic conditions among the poor and those with limited access to services for diagnosis and treatment. It is therefore possible that self-reported NCDs underestimate true prevalence and this can also explain why chronic conditions were more common in urban residents in the study samples [56].

It must be acknowledged that the contribution of each individual factor in a decomposition changes with the addition of new variables and therefore the reported contributions by the determinants are sensitive to the number of variables included in the regression models. We also acknowledge that measuring inequalities in disability in terms of a priori groups as we have done assumes that the groups are meaningful. There may be noise in the urban vs. rural classification. This may also be a reason for the larger proportions of unexplained inequality in the residence decomposition.

### Public health implications

Strategies that target improving education and employment opportunities in women and rural residents are one way of reducing inequalities shaped by gender, particularly in India but also in China. However, it needs to be acknowledged that gender inequalities are entrenched and require major long term structural, cultural and social change. Chronic conditions also contributed to the inequality between men and women, particularly in China. Prevention and early treatment efforts to mitigate chronic conditions in these two countries are essential steps for preventing premature mortality related to chronic diseases, as well for promoting good health and quality of life in older adults.

## Conclusions

This study identifies some of the likely determinants of disability among older adults, and their policy challenges, for India and China. Gender and location are important determinants of disability in older adults in both China and India. Attempts to slow disability and promote good health in these two populous rapidly ageing countries should focus on social determinants, such as education, and employment, and on alleviating or preventing chronic conditions. In particular, women in both countries and rural residents deserve to be targeted in the promotion of good health and quality of life in later adult years of life. Health policies and interventions require appropriate data from studies such as this. There is a need for further research, using both qualitative and quantitative methods, to question and challenge entrenched practices and institutions and grasp the implications of global economic and social changes that are impacting on population health and ageing in China and India.

## Additional files

Additional file 1: Appendix B. This file includes the questions about health and functioning used in calculating the disability score (PDF 84 kb)

Additional file 2: Appendix A. This file describes the statistical steps used in calculating the disability score. (PDF 83 kb)

Additional file 3: Appendix C. This file outlines the Blinder-Oaxaca method of decomposition. (PDF 265 kb)

## Abbreviations

C: Coefficient
CI: Confidence interval
ICF: International Classification of Functioning
IRT: Item Response Theory
NCDs: Non-communicable diseases
SAGE: Study on global AGEing and adult health
VIF: Variance inflation factor
WHO: World Health Organization
