# Supplementary material for: Disability and ageing in China and India – decomposing the effects of gender and residence. Results from the WHO study on global AGEing and adult health (SAGE)
Source: BMC Geriatr. 2017 Aug 31;17:197. doi: 10.1186/s12877-017-0589-y (PMC5579922; doi:10.1186/s12877-017-0589-y)
Supplement: Supplementary file 1 — This file includes the questions about health and functioning used in calculating the disability score (PDF 84 kb) [file 12877_2017_589_MOESM1_ESM.pdf]

Appendix B

Section 2000: Health State Descriptions

Time Begin ☐☐:☐☐

Now we will switch to questions specifically about your health. The first questions are about your overall health, including both your physical and your mental health. By difficulty in the second question, I mean requiring increased effort, discomfort or pain, slowness or changes in the way you do the activity.

Q2000	In general, how would you rate your health today?	1 Very good<br>2 Good<br>3 Moderate<br>4 Bad<br>5 Very bad
Q2001	Overall in the last 30 days, how much difficulty did you have with work or household activities?	1 None<br>2 Mild<br>3 Moderate<br>4 Severe<br>5 Extreme/cannot do

Now I would like to review the different functions of your body. When answering these questions, I would like you to think about the last 30 days, taking both good and bad days into account. When I ask about difficulty, I would like you to consider how much difficulty you have had, on average, in the last 30 days, while doing the activity in the way that you usually do it. Let me remind you, by difficulty I mean requiring increased effort, discomfort or pain, slowness or changes in the way you do the activity.

INTERVIEWER: Read and show scale to respondent.

MOBILITY

	Overall in the last 30 days, how much difficulty did you have …	NONE	MILD	MODERATE	SEVERE	EXTREME / CANNOT DO
Q2002	… with moving around?	1	2	3	4	5
Q2003	… in vigorous activities ('vigorous activities' require hard physical effort and cause large increases in breathing or heart rate)?	1	2	3	4	5

INTERVIEWER: Use Showcard if needed for mobility.

SELF-CARE

	Overall in the last 30 days, how much difficulty did you have …	NONE	MILD	MODERATE	SEVERE	EXTREME / CANNOT DO
Q2004	… with self-care, such as bathing/washing or dressing yourself?	1	2	3	4	5
Q2005	… in taking care of and maintaining your general appearance (for example, grooming, looking neat and tidy)?	1	2	3	4	5
Q2006	… in staying by yourself for a few days (3 to 7 days)?	1	2	3	4	5

PAIN AND DISCOMFORT

	Overall in the last 30 days,…	NONE	MILD	MODERATE	SEVERE	EXTREME / CANNOT DO
Q2007	…how much of bodily aches or pains did you have?	1	2	3	4	5
Q2008	…how much bodily discomfort did you have?	1	2	3	4	5
	If Q2007 and Q2008 are both = 1, "None"……➔					Q2010
Q2009	… how much difficulty did you have in your daily life because of your pain?	1	2	3	4	5

COGNITION

	Overall in the last 30 days, how much difficulty…	NONE	MILD	MODERATE	SEVERE	EXTREME / CANNOT DO
Q2010	… did you have with concentrating or remembering things?	1	2	3	4	5
Q2011	… did you have in learning a new task (for example, learning how to get to a new place, learning a new game, learning a new recipe)?	1	2	3	4	5

INTERPERSONAL ACTIVITIES

	Overall in the last 30 days, how much difficulty did you have,…	NONE	MILD	MODERATE	SEVERE	EXTREME / CANNOT DO
Q2012	… with personal relationships or participation in the community?	1	2	3	4	5
Q2013	… in dealing with conflicts and tensions with others?	1	2	3	4	5
Q2014	… with making new friendships or maintaining current friendships?	1	2	3	4	5
Q2015	…with dealing with strangers?	1	2	3	4	5

SLEEP AND ENERGY

	Overall in the last 30 days, how much of a problem did you…	NONE	MILD	MODERATE	SEVERE	EXTREME / CANNOT DO
Q2016	… have with sleeping, such as falling asleep, waking up frequently during the night or waking up too early in the morning?	1	2	3	4	5
Q2017	… have due to not feeling rested and refreshed during the day (for example, feeling tired, not having energy)?	1	2	3	4	5

AFFECT

	Overall in the last 30 days, how much of a problem did you have…	NONE	MILD	MODERATE	SEVERE	EXTREME / CANNOT DO
Q2018	…with feeling sad, low or depressed?	1	2	3	4	5
Q2019	… with worry or anxiety?	1	2	3	4	5

VISION (*Respondent should answer, as when wearing glasses/contact lenses if used*)

Q2020	When was the last time you had your eyes examined by a medical professional?<br>*INTERVIEWER: ENTER YEARS AGO. ENTER "00" IF LESS THAN 1 YEAR.*	☐☐ YEARS AGO<br>-8 *DON'T KNOW*<br>98 NEVER
Q2021	Do you use eyeglasses or contact lenses to see far away (for example, across the street)?	1 YES<br>2 NO
Q2022	Do you use eyeglasses or contact lenses to see up close (for example at arms length, like when you are reading)?	1 YES<br>2 NO
Q2023	In the last 30 days, how much difficulty did you have in seeing and recognising an object or a person you know across the road (from a distance of about 20 meters)?	1 NONE<br>2 MILD<br>3 MODERATE<br>4 SEVERE<br>5 EXTREME / CANNOT DO
Q2024	In the last 30 days, how much difficulty did you have in seeing and recognising an object at arm's length (for example, reading)?	1 NONE<br>2 MILD<br>3 MODERATE<br>4 SEVERE<br>5 EXTREME / CANNOT DO

FUNCTIONING ASSESSMENT

These next questions ask about difficulties due to health conditions. Health conditions include diseases or illnesses, other health problems that may be short or long lasting, injuries, mental or emotional problems, and problems with alcohol or drugs.

Think back over the last 30 days and answer these questions thinking about how much difficulty you had doing the following activities. Some of these questions may seem repetitive, but we do need your attention and it is important to give us answers to each question.

INTERVIEWER: *For each question, please circle only one response. 'N/A' means 'not applicable'.*

	In the last 30 days, how much difficulty did you have …	None	Mild	Moderate	Severe	Extreme/ cannot do	N/A
Q2025	… in sitting for long periods?	1	2	3	4	5	9
Q2026	… in walking 100 meters?	1	2	3	4	5	9
Q2027	… in standing up from sitting down?	1	2	3	4	5	9
Q2028	… in standing for long periods?	1	2	3	4	5	9
Q2029	… with climbing one flight of stairs without resting?	1	2	3	4	5	9
Q2030	… with stooping, kneeling or crouching?	1	2	3	4	5	9
Q2031	… picking up things with your fingers (such as picking up a coin from a table)?	1	2	3	4	5	9
Q2032	… in taking care of your household responsibilities?	1	2	3	4	5	9
