# Supplementary material for: Disability and ageing in China and India – decomposing the effects of gender and residence. Results from the WHO study on global AGEing and adult health (SAGE)
Source: BMC Geriatr. 2017 Aug 31;17:197. doi: 10.1186/s12877-017-0589-y (PMC5579922; doi:10.1186/s12877-017-0589-y)
Supplement: Supplementary file 2 — This file describes the statistical steps used in calculating the disability score. (PDF 83 kb) [file 12877_2017_589_MOESM2_ESM.pdf]

Appendix A

Calculating the disability score

The WHO-SAGE individual questionnaire includes 16 questions about health and functioning. See Appendix B. The questions cover eight domains of health-related function: vision; mobility; self-care; cognition; interpersonal activities; pain and discomfort; sleep and energy, and affect. Respondents rated the amount of difficulty they had experienced in each domain in the previous 30 days using the categories: no difficulty, mild difficulty, moderate difficulty, severe difficulty and extreme difficulty.

A composite score for each individual was calculated across all the 16 items to estimate where each individual in the survey would be placed on a latent dimension of functioning. An Item Response Theory (IRT) approach using a Rasch model was used to construct this score. Rasch models help to transform raw data from the categorical ordered self-report scale of difficulty to an equal-interval scale. Equality of intervals is achieved through log transformations of raw data odds, and abstraction is accomplished through probabilistic equations. This transformation for the partial credit model allows not only for a hierarchical order of difficulty of the items but for different thresholds of item categories as well.

The original 16-item health module was analysed with the Rasch Rating Scale model using the WINSTEPS computer program. Surveys that used only 8 items and those that used the full 16 items were analysed together in this model to yield a common scale across all surveys. A calibration was obtained for each item. To determine how well each item contributed to the common global functioning measurement, chi squared (χ^2) goodness-of-fit statistics, known as Infit Mean Squares (MNSQ), were also calculated. The Infit MNSQ

ranged from 0.77 to 1.38 (SD = 0.27). Only the domain of vision slightly exceeded the recommended item misfit threshold of 1.3, but this domain was retained in the analysis. The Dimensionality Map – a principal components factor plot on the residuals – showed no existence of a secondary factor. To test Differential Item Functioning (DIF) by country, a logistic-regression approach was used. The pseudo-R2 change of 0.02 showed a tolerable DIF effect. Finally, to take into account each particular item calibration for the 16 health items, raw scores were transformed through Rasch modelling into a new scale of scores, with 0 = no difficulty and 100 = complete difficulty (1).

1. World Health Organization, The World Bank. World report on disability. http://www.who.int/disabilities/world_report/2011/en/: WHO 2011.
