# Supplementary material for: Disability and ageing in China and India – decomposing the effects of gender and residence. Results from the WHO study on global AGEing and adult health (SAGE)
Source: BMC Geriatr. 2017 Aug 31;17:197. doi: 10.1186/s12877-017-0589-y (PMC5579922; doi:10.1186/s12877-017-0589-y)
Supplement: Supplementary file 3 — This file outlines the Blinder-Oaxaca method of decomposition. (PDF 265 kb) [file 12877_2017_589_MOESM3_ESM.pdf]

Appendix C

The Stata Journal (2008)
8, Number 4, pp. 453–479

The Blinder–Oaxaca decomposition for linear regression models

Ben Jann
ETH Zürich
Zürich, Switzerland
jann@soz.gess.ethz.ch

Abstract. The counterfactual decomposition technique popularized by Blinder (1973, *Journal of Human Resources*, 436–455) and Oaxaca (1973, *International Economic Review*, 693–709) is widely used to study mean outcome differences between groups. For example, the technique is often used to analyze wage gaps by sex or race. This article summarizes the technique and addresses several complications, such as the identification of effects of categorical predictors in the detailed decomposition or the estimation of standard errors. A new command called `oaxaca` is introduced, and examples illustrating its usage are given.

Keywords: st0151, oaxaca, Blinder–Oaxaca decomposition, outcome differential, wage gap

1 Introduction

An often used methodology to study labor-market outcomes by groups (sex, race, and so on) is to decompose mean differences in log wages based on linear regression models in a counterfactual manner. The procedure is known in the literature as the Blinder–Oaxaca decomposition (Blinder 1973; Oaxaca 1973). It divides the wage differential between two groups into a part that is "explained" by group differences in productivity characteristics, such as education or work experience, and a residual part that cannot be accounted for by such differences in wage determinants. This "unexplained" part is often used as a measure for discrimination, but it also subsumes the effects of group differences in unobserved predictors. Most applications of the technique can be found in the labor market and discrimination literature (for meta studies, see, e.g., Stanley and Jarrell [1998] or Weichselbaumer and Winter-Ebmer [2005]). However, the method can also be useful in other fields. In general, the technique can be employed to study group differences in any (continuous and unbounded[1]) outcome variable. For example, O'Donnell et al. (2008) use it to analyze health inequalities by poverty status.

The purpose of this article is to introduce a new Stata command, called `oaxaca`, that implements the Blinder–Oaxaca decomposition. In the next section, the most common variants of the decomposition are summarized, and a number of issues, such as the identification of the contribution of categorical predictors or the estimation of standard errors, are addressed. The third section then describes the syntax and options of the

1. See Sinning, Hahn, and Bauer (in this issue) for the decomposition of group differences in categorical or bounded outcomes.

© 2008 StataCorp LP st0151

new `oaxaca` command, and the fourth section uses labor-market data to illustrate its applications.

2 Methods and formulas

Given are two groups, A and B; an outcome variable, Y; and a set of predictors. For example, think of a group of males and a group of females, (log) wages as the outcome variable, and human capital indicators such as education and work experience as predictors. The question now is how much of the mean outcome difference,

$$R = E(Y_A) - E(Y_B)$$

where $E(Y)$ denotes the expected value of the outcome variable, is accounted for by group differences in the predictors.

Based on the linear model

$$Y_\ell = X'_\ell \beta_\ell + \epsilon_\ell, \quad E(\epsilon_\ell) = 0 \quad \ell \in (A, B)$$

where X is a vector containing the predictors and a constant, β contains the slope parameters and the intercept, and ϵ is the error, the mean outcome difference can be expressed as the difference in the linear prediction at the group-specific means of the regressors. That is,

$$R = E(Y_A) - E(Y_B) = E(X_A)'\beta_A - E(X_B)'\beta_B \tag{1}$$

because

$$E(Y_\ell) = E(X'_\ell \beta_\ell + \epsilon_\ell) = E(X'_\ell \beta_\ell) + E(\epsilon_\ell) = E(X_\ell)'\beta_\ell$$

where $E(\beta_\ell) = \beta_\ell$ and $E(\epsilon_\ell) = 0$ by assumption.

To identify the contribution of group differences in predictors to the overall outcome difference, (1) can be rearranged, for example, as follows (see Winsborough and Dickinson [1971]; Jones and Kelley [1984]; and Daymont and Andrisani [1984]):

$$R = \{E(X_A) - E(X_B)\}' \beta_B + E(X_B)'(\beta_A - \beta_B) + \{E(X_A) - E(X_B)\}' (\beta_A - \beta_B) \tag{2}$$

This is a "threefold" decomposition; that is, the outcome difference is divided into three components:

$$R = E + C + I$$

The first component,

$$E = \{E(X_A) - E(X_B)\}' \beta_B$$

amounts to the part of the differential that is due to group differences in the predictors (the "endowments effect"). The second component,

$$C = E(X_B)'(\beta_A - \beta_B)$$

measures the contribution of differences in the coefficients (including differences in the intercept). And the third component,

$$I = \{E(X_A) - E(X_B)\}' (\beta_A - \beta_B)$$

is an interaction term accounting for the fact that differences in endowments and coefficients exist simultaneously between the two groups.

The decomposition shown in (2) is formulated from the viewpoint of group B. That is, the group differences in the predictors are weighted by the coefficients of group B to determine the endowments effect (E). The E component measures the expected change in group B's mean outcome if group B had group A's predictor levels. Similarly, for the C component (the "coefficients effect"), the differences in coefficients are weighted by group B's predictor levels. That is, the C component measures the expected change in group B's mean outcome if group B had group A's coefficients. Naturally, the differential can also be expressed from the viewpoint of group A, yielding the reverse threefold decomposition,

$$R = \{E(X_A) - E(X_B)\}' \beta_A + E(X_A)'(\beta_A - \beta_B) - \{E(X_A) - E(X_B)\}' (\beta_A - \beta_B) \quad (3)$$

Now the endowments effect amounts to the expected change of group A's mean outcome if group A had group B's predictor levels. The coefficients effect quantifies the expected change in group A's mean outcome if group A had group B's coefficients.

An alternative decomposition prominent in the discrimination literature results from the concept that there is a nondiscriminatory coefficient vector that should be used to determine the contribution of the differences in the predictors. Let β^* be such a nondiscriminatory coefficient vector. The outcome difference can then be written as

$$R = \{E(X_A) - E(X_B)\}' \beta^* + \{E(X_A)'(\beta_A - \beta^*) + E(X_B)'(\beta^* - \beta_B)\} \quad (4)$$

We now have a "twofold" decomposition,

$$R = Q + U$$

where the first component,

$$Q = \{E(X_A) - E(X_B)\}' \beta^*$$

is the part of the outcome differential that is explained by group differences in the predictors (the "quantity effect"), and the second component,

$$U = E(X_A)'(\beta_A - \beta^*) + E(X_B)'(\beta^* - \beta_B)$$

is the unexplained part. The latter is usually attributed to discrimination, but it is important to recognize that it also captures all the potential effects of differences in unobserved variables.

The unexplained part in (4) is sometimes further decomposed. Let $\beta_A = \beta^* + \delta_A$ and $\beta_B = \beta^* + \delta_B$, with δ_A and δ_B as group-specific discrimination parameter vectors (positive or negative discrimination, depending on the sign). U can then be expressed as

$$U = E(X_A)'\delta_A - E(X_B)'\delta_B$$

That is, the unexplained component of the differential can be subdivided into a part,

$$U_A = E(X_A)'\delta_A$$

that measures discrimination in favor of group A and a part,

$$U_B = -E(X_B)'\delta_B$$

that quantifies discrimination against group B.[2] Again, however, this interpretation hinges on the assumption that there are no relevant unobserved predictors.

The estimation of the components of the threefold decompositions shown in (2) and (3) is straightforward. Let $\widehat{\beta}_A$ and $\widehat{\beta}_B$ be the least-squares estimates for β_A and β_B, obtained separately from the two group-specific samples. Furthermore, use the group means $\overline{X}_A$ and $\overline{X}_B$, as estimates for $E(X_A)$ and $E(X_B)$. Based on these estimates, (2) and (3) are computed as

$$\widehat{R} = \overline{Y}_A - \overline{Y}_B = (\overline{X}_A - \overline{X}_B)'\widehat{\beta}_B + \overline{X}'_B(\widehat{\beta}_A - \widehat{\beta}_B) + (\overline{X}_A - \overline{X}_B)'(\widehat{\beta}_A - \widehat{\beta}_B)$$

and

$$\widehat{R} = \overline{Y}_A - \overline{Y}_B = (\overline{X}_A - \overline{X}_B)'\widehat{\beta}_A + \overline{X}'_A(\widehat{\beta}_A - \widehat{\beta}_B) - (\overline{X}_A - \overline{X}_B)'(\widehat{\beta}_A - \widehat{\beta}_B)$$

The determination of the components of the twofold decomposition shown in (4) is more involved because an estimate for the unknown nondiscriminatory coefficients vector β^* is needed. Several suggestions have been made in the literature. For example, there may be reason to assume that discrimination is directed toward only one of the groups, so that $\beta^* = \beta_A$ or $\beta^* = \beta_B$ (see Oaxaca [1973], who speaks of an "index number problem"). Again assume that members of group A are males and members of group B are females. If, for instance, wage discrimination is directed only against women and there is no (positive) discrimination of men, then we can use $\widehat{\beta}_A$ as an estimate for β^* and compute (4) as

$$\widehat{R} = (\overline{X}_A - \overline{X}_B)'\widehat{\beta}_A + \overline{X}'_B(\widehat{\beta}_A - \widehat{\beta}_B) \tag{5}$$

Similarly, if there is only (positive) discrimination of men but no discrimination of women, the decomposition is

$$\widehat{R} = (\overline{X}_A - \overline{X}_B)'\widehat{\beta}_B + \overline{X}'_A(\widehat{\beta}_A - \widehat{\beta}_B) \tag{6}$$

Often, however, there is no specific reason to assume that the coefficients of one or the other group are nondiscriminating. Moreover, economists have argued that the

2. U_A and U_B have opposite interpretations. A positive value for U_A reflects positive discrimination of group A; a positive value for U_B indicates negative discrimination of group B.

undervaluation of one group comes along with an overvaluation of the other (e.g., Cotton [1988]). Reimers (1983) therefore proposes using the average coefficients over both groups as an estimate for the nondiscriminatory parameter vector; that is,

$$\widehat{\beta}^* = 0.5\widehat{\beta}_A + 0.5\widehat{\beta}_B$$

Similarly, Cotton (1988) suggests to weight the coefficients by the group sizes, n_A and n_B; that is,

$$\widehat{\beta}^* = \frac{n_A}{n_A + n_B}\widehat{\beta}_A + \frac{n_B}{n_A + n_B}\widehat{\beta}_B$$

Furthermore, based on theoretical derivations, Neumark (1988) advocates the use of the coefficients from a pooled regression over both groups as an estimate for β^*.

As pointed out by Oaxaca and Ransom (1994) and others, (4) can also be expressed as

$$\begin{aligned} R = & \{E(X_A) - E(X_B)\}' \{\mathbf{W}\beta_A + (\mathbf{I} - \mathbf{W})\beta_B\} \\ & + \{(\mathbf{I} - \mathbf{W})'E(X_A) + \mathbf{W}'E(X_B)\}' (\beta_A - \beta_B) \end{aligned}$$

where $\mathbf{W}$ is a matrix of relative weights given to the coefficients of group A, and $\mathbf{I}$ is the identity matrix. For example, choosing $\mathbf{W} = \mathbf{I}$ is equivalent to setting $\beta^* = \beta_A$. Similarly, $\mathbf{W} = 0.5\mathbf{I}$ is equivalent to $\beta^* = 0.5\beta_A + 0.5\beta_B$. Furthermore, Oaxaca and Ransom (1994) show that

$$\widehat{\mathbf{W}} = \Omega = (\mathbf{X}_A'\mathbf{X}_A + \mathbf{X}_B'\mathbf{X}_B)^{-1}\mathbf{X}_A'\mathbf{X}_A \tag{7}$$

with $\mathbf{X}$ as the observed data matrix is equivalent to using the coefficients from a pooled model over both groups as the reference coefficients.[3]

An issue with the approach by Neumark (1988) and Oaxaca and Ransom (1994) is that it can inappropriately transfer some of the unexplained parts of the differential into the explained component, although this does not seem to have received much attention in the literature.[4] Assume a simple model of log wages ($\ln W$) on education (Z) with the sex-specific intercepts α_M and α_F due to discrimination. The model is

$$\ln W = \begin{cases} \alpha_M + \gamma Z + \epsilon, & \text{if “male”} \\ \alpha_F + \gamma Z + \epsilon, & \text{if “female”} \end{cases}$$

3. Another solution is to set $\mathbf{W} = \text{diag}(\beta - \beta_B) \times \text{diag}(\beta_A - \beta_B)^{-1}$, where β without a subscript denotes the coefficients from the pooled model. Although the decomposition results are the same, this approach yields a weighting matrix that is quite different from Oaxaca and Ransom's (1994) Ω. For example, whereas $\mathbf{W}$ computed as described in this footnote is a diagonal matrix, Ω has off-diagonal elements that are unequal to zero and are not even symmetric.
4. An exception is Fortin (2006).

Let $\alpha_M = \alpha$ and $\alpha_F = \alpha + \delta$, where δ is the discrimination parameter. Then the model can also be expressed as

$$\ln W = \alpha + \gamma Z + \delta F + \epsilon$$

with F as an indicator for "female". Assume that $\gamma > 0$ (positive relation between education and wages) and $\delta < 0$ (discrimination against women). If we use γ^* from a pooled model,

$$\ln W = \alpha^* + \gamma^* Z + \epsilon^*$$

in (4), then following from the theory on omitted variables (see, e.g., Gujarati [2003, 510–513]), the explained part of the differential is

$$Q = \{E(Z_M) - E(Z_F)\}\, \gamma^* = \{E(Z_M) - E(Z_F)\} \left\{ \gamma + \delta \frac{\text{Cov}(Z, G)}{\text{Var}(Z)} \right\}$$

where $\text{Var}(Z)$ is the variance of Z, and $\text{Cov}(Z, G)$ is the covariance between Z and G. If men on average are better educated than women, then the covariance between Z and G is negative, and the explained part of the decomposition gets overstated (given $\gamma > 0$ and $\delta < 0$). In essence, the difference in wages between men and women is explained by sex.

To avoid such a distortion of the decomposition results because of the residual group difference spilling over into the slope parameters of the pooled model, my recommendation is to always include a group indicator in the pooled model as an additional covariate.

Estimation of sampling variances

Given the popularity of the Blinder–Oaxaca procedure, it is astonishing how little attention has been paid to the issue of statistical inference. Most studies in which the procedure is applied only report point estimates for the decomposition results and do not make any indications about sampling variances or standard errors.[5] However, for an adequate interpretation of the results, approximate measures of statistical precision are indispensable.

Approximate variance estimators for certain variants of the decomposition were first proposed by Oaxaca and Ransom (1998), with Greene (2008, 55–56) making similar suggestions. The estimators by Oaxaca and Ransom (1998) and Greene (2008) are a good starting point, but they neglect an important source of variation. Most social-science studies on discrimination are based on survey data where all (or most of) the variables are random variables. That is, not only the outcome variable but also the predictors are subject to sampling variation (an exception would be experimental factors set by the researcher). Whereas an important result for regression analysis is that it does not matter for the variance estimates whether regressors are stochastic or fixed, this is

5. Exceptions are, for example, Oaxaca and Ransom (1994, 1998), Silber and Weber (1999), Horrace and Oaxaca (2001), Fortin (2006), Heinrichs and Kennedy (2007), and Lin (2007). Furthermore, Jackson and Lindley (1989) and Shrestha and Sakellariou (1996) propose statistical tests for discrimination.

not true for the Blinder–Oaxaca decomposition. The decomposition is based on multiplying regression coefficients by means of regressors. If the regressors are stochastic, then the means have sampling variances. These variances are of the same asymptotic order as the variances of the coefficients (think of the means as the intercepts from regression models without covariates). To get consistent standard errors for the decomposition results, it seems important to take into account the variability induced by the randomness of the predictors.

Consider the expression

$$\overline{Y} = \overline{X}'\widehat{\beta} \tag{8}$$

where $\overline{X}$ is the vector of mean estimates for the predictors, and $\widehat{\beta}$ contains the least-squares estimates of the regression coefficients. If the predictors are stochastic, then $\overline{X}$ and $\widehat{\beta}$ are both subject to sampling variation. Assuming that $\overline{X}$ and $\widehat{\beta}$ are uncorrelated (which follows from the standard regression assumption that the conditional expectation of the error is zero for all covariate values; of course, this is only true if the model is correctly specified), the variance of (8) can be written as

$$V(\overline{X}'\widehat{\beta}) = E(\overline{X})'V(\widehat{\beta})E(\overline{X}) + E(\widehat{\beta})'V(\overline{X})E(\widehat{\beta}) + \text{trace}\left\{V(\overline{X})V(\widehat{\beta})\right\}$$

where $V(\overline{X})$ and $V(\widehat{\beta})$ are the variance–covariance matrices for $\overline{X}$ and $\widehat{\beta}$ (see the proof in Jann [2005b]; for the variance of the product of two independent random variables, also see Mood, Graybill, and Boes [1974, 180]). By inserting estimates for the expectations and variance matrices, we get the variance estimator

$$\widehat{V}(\overline{X}'\widehat{\beta}) = \overline{X}'\widehat{V}(\widehat{\beta})\overline{X} + \widehat{\beta}'\widehat{V}(\overline{X})\widehat{\beta} + \text{trace}\left\{\widehat{V}(\overline{X})\widehat{V}(\widehat{\beta})\right\} \tag{9}$$

$\widehat{V}(\widehat{\beta})$ is simply the variance–covariance matrix obtained from the regression procedure. A natural estimator for $V(\overline{X})$ is $\widehat{V}(\overline{X}) = \mathcal{X}'\mathcal{X}/\{n(n-1)\}$, where $\mathcal{X}$ is the centered-data matrix, i.e., $\mathcal{X} = \mathbf{X} - \mathbf{1}\overline{X}'$.

The variances for the components of the Blinder–Oaxaca decomposition can be derived analogously. For example, ignoring the asymptotically vanishing[6] last term in (9) and assuming that the two groups are independent, the approximate variance estimators for the two terms of the decomposition shown in (5) are

$$\widehat{V}\{(\overline{X}_A - \overline{X}_B)'\widehat{\beta}_A\} \approx (\overline{X}_A - \overline{X}_B)'\widehat{V}(\widehat{\beta}_A)(\overline{X}_A - \overline{X}_B) + \widehat{\beta}_A'\left\{\widehat{V}(\overline{X}_A) + \widehat{V}(\overline{X}_B)\right\}\widehat{\beta}_A \tag{10}$$

and

$$\widehat{V}\{\overline{X}_B'(\widehat{\beta}_A - \widehat{\beta}_B)\} \approx \overline{X}_B'\left\{\widehat{V}(\widehat{\beta}_A) + \widehat{V}(\widehat{\beta}_B)\right\}\overline{X}_B + (\widehat{\beta}_A - \widehat{\beta}_B)'\widehat{V}(\overline{X}_B)(\widehat{\beta}_A - \widehat{\beta}_B) \tag{11}$$

where we make use of the fact that the variance of the sum of two uncorrelated random variables is equal to the sum of the individual variances. An interesting point about

6. Whereas the first and second terms are of the order $O(n^{-1})$, the last term is $O(n^{-2})$.

(10) and (11) is that ignoring the stochastic nature of the predictors will primarily affect the variance of the first term of the decomposition (the explained part). This is because in most applications group differences in coefficients and means are much smaller than the levels of coefficients and means.

It is possible to develop similar formulas for all the decomposition variants outlined above, but derivations can get complicated once a pooled model is used and covariances between the pooled model and the group models have to be taken into account. Likewise, derivations can get complicated if the assumption of independence between the two groups is loosened (e.g., if dealing with a cluster sample). An alternative approach that is simple and general and produces equivalent results is to estimate the joint variance–covariance matrix of all used statistics (see Weesie [1999] and [R] **suest**) and then apply the "delta method" (see [R] **nlcom** and the references therein). In fact, for independence between the two groups, the results of the delta method for (2) are formally equal to (10) and (11). Furthermore, a general result for the delta method is that if the input variance matrix is asymptotically normal, then the variance matrix of the transformed statistics is asymptotically normal (see, e.g., Greene [2008, 68–71]). That is, because asymptotic normality holds for regression coefficients and mean estimates under very general conditions, the variances obtained by the delta method can be used to construct approximate confidence intervals for the decomposition results in the usual manner.

Detailed decomposition

Often, not only is the total decomposition of the outcome differential into an explained and an unexplained part of interest, but also the detailed contributions of the single predictors or sets of predictors are subject to investigation. For example, one might want to evaluate how much of the gender wage gap is due to differences in education and how much is due to differences in work experience. Similarly, it might be informative to determine how much of the unexplained gap is related to differing returns to education and how much is related to differing returns to work experience.

Identifying the contributions of the individual predictors to the explained part of the differential is easy because the total component is a simple sum over the individual contributions. For example, in (5),

$$\widehat{Q} = (\overline{X}_A - \overline{X}_B)'\widehat{\beta}_A = (\overline{X}_{1A} - \overline{X}_{1B})\widehat{\beta}_{1A} + (\overline{X}_{2A} - \overline{X}_{2B})\widehat{\beta}_{2A} + \cdots$$

where $\overline{X}_1, \overline{X}_2, \ldots$ are the means of the single regressors, and $\widehat{\beta}_1, \widehat{\beta}_2, \ldots$ are the associated coefficients. The first summand reflects the contribution of the group differences in $\overline{X}_1$; the second, of differences in $\overline{X}_2$; and so on. Also the estimation of standard errors for the individual contributions is straightforward.

Similarly, using (5) as an example, the individual contributions to the unexplained part are the summands in

$$\widehat{U} = \overline{X}_B'(\widehat{\beta}_A - \widehat{\beta}_B) = \overline{X}_{1B}'(\widehat{\beta}_{1A} - \widehat{\beta}_{1B}) + \overline{X}_{2B}'(\widehat{\beta}_{2A} - \widehat{\beta}_{2B}) + \cdots$$

However, other than for the explained part of the decomposition, the contributions to the unexplained part can depend on arbitrary scaling decisions if the predictors do not have natural zero points (e.g., Jones and Kelley [1984, 334]). Without loss of generality, assume a simple model with just one explanatory variable:

$$Y_\ell = \beta_{0\ell} + \beta_{1\ell} Z_\ell + \epsilon_\ell, \quad \ell \in (A, B)$$

The unexplained part of the decomposition based on (5) then is

$$\widehat{U} = (\widehat{\beta}_{0A} - \widehat{\beta}_{0B}) + (\widehat{\beta}_{1A} - \widehat{\beta}_{1B})\overline{Z}_B$$

The first summand is the part of the unexplained gap that is due to "group membership" (Jones and Kelley 1984); the second summand reflects the contribution of differing returns to Z. Now assume that the zero point of Z is shifted by adding a constant, a. The effect of such a shift on the decomposition results is as follows:

$$\widehat{U} = \left\{(\widehat{\beta}_{0A} - a\widehat{\beta}_{1A}) - (\widehat{\beta}_{0B} - a\widehat{\beta}_{1B})\right\} + (\widehat{\beta}_{1A} - \widehat{\beta}_{1B})(\overline{Z}_B + a)$$

Evidently, the scale shift changes the results; a portion amounting to $a(\widehat{\beta}_{1A} - \widehat{\beta}_{1B})$ is transferred from the group membership component to the part that is due to different slope coefficients. The conclusion is that the detailed decomposition results for the unexplained part have a meaningful interpretation only for variables for which scale shifts are not allowed, that is, for variables that have a natural zero point.[7]

A related issue that has received much attention in the literature is that the decomposition results for categorical predictors depend on the choice of the omitted base category (Jones 1983; Jones and Kelley 1984; Oaxaca and Ransom 1999; Nielsen 2000; Horrace and Oaxaca 2001; Gardeazabal and Ugidos 2004; Polavieja 2005; Yun 2005b). The effect of a categorical variable is usually modeled by including 0/1 variables ("dummy" variables) for the different categories in the regression equation, where one of the categories (the "base" category) is omitted to avoid collinearity. It is easy to see that the decomposition results for the single 0/1 variables depend on the choice of the base category, because the associated coefficients quantify differences with respect to the base category. If the base category changes, the decomposition results change.

For the explained part of the decomposition, this may not be critical because the sum of the contributions of the single indicator variables (that is, the total contribution of the categorical variable) is unaffected by the choice of the base category. For the unexplained part of the decomposition, however, there is again a tradeoff between the group membership component (the difference in intercepts) and the part attributed

7. The problem does not occur for the explained part of the decomposition or the interaction component in the threefold decomposition because a cancels out in these cases. Furthermore, stretching or compressing the scales of the X variables (multiplication by a constant) does not alter any of the decomposition results because such multiplicative transformations are counterbalanced by the coefficient estimates.

to differences in slope coefficients. For the unexplained part, changing the base category not only alters the results for the single dummy variables but also changes the contribution of the categorical variable as a whole.

An intuitively appealing solution to the problem has been proposed by Gardeazabal and Ugidos (2004) and Yun (2005b). The idea is to restrict the coefficients for the single categories to sum to zero, that is, to express effects as deviations from the grand mean. This can be implemented by restricted least-squares estimation or by transforming the dummy variables before model estimation, as proposed by Gardeazabal and Ugidos (2004).[8] A more convenient method in the context of the Blinder–Oaxaca decomposition is to estimate the group models by using the standard dummy coding and then transform the coefficient vectors so that deviations from the grand mean are expressed and the (redundant) coefficient for the base category is added (Suits 1984; Yun 2005b). If applied to such transformed estimates, the results of the Blinder–Oaxaca decomposition are independent of the choice of the omitted category. Furthermore, the results are equal to the simple averages of the results one would get from a series of decompositions in which the categories are used one after another as the base category (Yun 2005b).

The deviation contrast transform works as follows. Given is the model

$$Y = \beta_0 + \beta_1 D_1 + \cdots + \beta_{k-1} D_{k-1} + \epsilon$$

where β_0 is the intercept, and D_j, $j = 1, \ldots, k-1$, are the dummy variables representing a categorical variable with k categories. Category k is the base category. Alternatively, the model can be formulated as

$$Y = \beta_0 + \beta_1 D_1 + \cdots + \beta_{k-1} D_{k-1} + \beta_k D_k + \epsilon$$

where β_k is constrained to zero. Now let

$$c = (\beta_1 + \cdots + \beta_k)/k$$

and define

$$\widetilde{\beta}_0 = \beta_0 + c \quad \text{and} \quad \widetilde{\beta}_j = \beta_j - c, \quad j = 1, \ldots, k$$

The transformed model is then

$$Y = \widetilde{\beta}_0 + \widetilde{\beta}_1 D_1 + \cdots + \widetilde{\beta}_k D_k + \epsilon, \quad \sum_{j=1}^{k} \widetilde{\beta}_j = 0$$

The transformed model is mathematically equivalent to the untransformed model. For example, the two models produce identical predictions. The variance–covariance matrix for the transformed model can be obtained by applying the general formula for weighted sums of random variables given in, e.g., Mood, Graybill, and Boes (1974, 179). Models with several sets of dummy variables can be transformed by applying the formulas to each set separately. Furthermore, the transformation can be applied to the interaction

8. In fact, the approach by Gardeazabal and Ugidos (2004) is simply what is known as the "effects coding" (Hardy 1993, 64–71) or the "deviation contrast coding" (Hendrickx 1999) approach.

terms between a categorical and a continuous variable in an analogous manner except that now c is added to the main effect of the continuous variable instead of the intercept. The application of the transform is not restricted to linear regression. It can be used with any model as long as the effects of the dummies are expressed as additive effects.

Other restrictions to identify the contribution of a categorical variable to the unexplained part of the decomposition are imaginable. For example, the restriction could be

$$\sum_{j=1}^{k} w_j \widetilde{\beta}_j = 0$$

where w_j are weights proportional to the relative frequencies of the categories, so the coefficients reflect deviations from the overall sample mean (Kennedy 1986; Haisken-DeNew and Schmidt 1997). Hence, there is still some arbitrariness in the method by Gardeazabal and Ugidos (2004) and Yun (2005b).

3 The oaxaca command

The methods presented above are implemented with a new command called `oaxaca`. The command first estimates the group models and possibly a pooled model over both groups using `regress` ([R] **regress**) or any user-specified estimation command. `suest` ([R] **suest**) is then applied, if necessary, to determine the combined variance–covariance matrix of the models, and the group means of the predictors are estimated by using `mean` ([R] **mean**). Finally, the various decomposition results and their standard errors (and covariances) are computed based on the combined parameter vector and variance–covariance matrix of the models' coefficients and the mean estimates.[9] The standard errors are obtained by the delta method.[10]

9. The covariances between the models' coefficients and the mean estimates are assumed to be zero in any case. This assumption can be violated in misspecified models.
10. `nlcom` ([R] **nlcom**) could be used to compute the variance–covariance matrix of the decomposition results. However, `nlcom` employs general methods based on numerical derivatives and is slow if the models contain many covariates. `oaxaca` therefore has its own specific implementation of the delta method based on analytic derivatives.

3.1 Syntax

The syntax of the `oaxaca` command is

`oaxaca` *depvar* [*indepvars*] [*if*] [*in*] [*weight*] `,` `by(`*groupvar*`)` [`swap` `detail`[`(`*dlist*`)`] `adjust(`*varlist*`)` `threefold`[`(reverse)`] `weight(`# [#...]`)` `pooled`[`(`*model_opts*`)`] `omega`[`(`*model_opts*`)`] `reference(`*name*`)` `split` `x1(`*names_and_values*`)` `x2(`*names_and_values*`)` `categorical(`*clist*`)` `svy`[`(`[*vcetype*] [`,` *svy_options*]`)`] `vce(`*vcetype*`)` `cluster(`*varname*`)` `fixed`[`(`*varlist*`)`] [`no`]`suest` `nose` `model1(`*model_opts*`)` `model2(`*model_opts*`)` `noisily` `xb` `level(`#`)` `eform` `nolegend`]

where *depvar* is the outcome variable of interest (e.g., log wages) and *indepvars* are predictors (e.g., education, work experience). *groupvar* identifies the groups to be compared. `oaxaca` typed without arguments replays the last results.

`fweights`, `aweights`, `pweights`, and `iweights` are allowed; see [U] **11.1.6 weight**. Furthermore, `bootstrap`, `by`, `jackknife`, `statsby`, and `xi` are allowed; see [U] **11.1.10 prefix, commands**. Weights are not allowed with the `bootstrap` prefix, and `aweights` are not allowed with the `jackknife` prefix. `vce()`, `cluster()`, and weights are not allowed with the `svy` option.

3.2 Options

Main

`by(`*groupvar*`)` specifies the *groupvar* that defines the two groups to be compared. `by()` is required.

`swap` reverses the order of the groups.

`detail`[`(`*dlist*`)`] specifies that the detailed results for the individual predictors be reported. Use *dlist* to subsume the results for sets of regressors (results for variables not appearing in *dlist* are listed individually). The syntax for *dlist* is

name`:` *varlist* [`,` *name*`:` *varlist* ...]

The usual shorthand conventions apply to the varlists specified in *dlist* (see `help varlist`; additionally, `_cons` is allowed). For example, specify `detail(exp:exp*)` to subsume `exp` (experience) and `exp2` (experience squared). *name* is any valid Stata name; it labels the set.

`adjust(`*varlist*`)` causes the differential to be adjusted by the contribution of the specified variables before performing the decomposition. This is useful, for example, if the specified variables are selection terms. `adjust()` is not needed for `heckman` models.

Decomposition type

`threefold`[`(reverse)`] computes the threefold decomposition. This is the default unless `weight()`, `pooled`, `omega`, or `reference()` is specified. The decomposition is expressed from the viewpoint of group 2 (B). Specify `threefold(reverse)` to express the decomposition from the viewpoint of group 1 (A).

`weight(`#[# `...`]`)` computes the twofold decomposition, where # [# `...`] are the weights given to group 1 (A) relative to group 2 (B) in determining the reference coefficients (weights are recycled if there are more coefficients than weights). For example, `weight(1)` uses the group 1 coefficients as the reference coefficients, and `weight(0)` uses the group 2 coefficients.

`pooled`[`(`*model_opts*`)`] computes the twofold decomposition by using the coefficients from a pooled model over both groups as the reference coefficients. *groupvar* is included in the pooled model as an additional control variable. Estimation details can be specified in parentheses; see the `model1()` option below.

`omega`[`(`*model_opts*`)`] computes the twofold decomposition by using the coefficients from a pooled model over both groups as the reference coefficients (excluding *groupvar* as a control variable in the pooled model). Estimation details can be specified in parentheses; see the `model1()` option below.

`reference(`*name*`)` computes the twofold decomposition by using the coefficients from a stored model. *name* is the name under which the model was stored; see [R] **estimates store**. Do not combine the `reference()` option with the bootstrap or jackknife methods.

`split` causes the unexplained component in the twofold decomposition to be split into a part related to group 1 (A) and a part related to group 2 (B). `split` is effective only if specified with `weight()`, `pooled`, `omega`, or `reference()`.

Only one of `threefold`, `weight()`, `pooled`, `omega`, and `reference()` is allowed.

X-values

`x1(`*names_and_values*`)` and `x2(`*names_and_values*`)` provide custom values for specific predictors to be used for group 1 (A) and group 2 (B) in the decomposition. The default is to use the group means of the predictors. The syntax for *names_and_values* is

varname [`=`] *value* [[`,`] *varname* [`=`] *value* ...]

For example, `x1(educ 12 exp 30)`.

`categorical(`*clist*`)` identifies sets of dummy variables representing categorical variables and transforms the coefficients so that the results of the decomposition are invariant to the choice of the (omitted) base category (deviation contrast transform). The syntax for *clist* is

varlist [`,` *varlist* ...]

Each varlist must contain a variable for the base category (that is, the base category indicator must exist in the data). The transform can also be applied to interactions between a categorical and a continuous variable. Specify the continuous variable in parentheses at the end of the list in this case, i.e.,

varlist `(`*varname*`)` [`,` ...]

and also include a list for the main effects. For example,

```
categorical(d1 d2 d3, xd1 xd2 xd3 (x))
```

where `x` is the continuous variable, and `d1`, `d2`, etc., and `xd1`, `xd2`, etc., are the main effects and interaction effects. The code for implementing the `categorical()` option has been taken from the user-written `devcon` command (Jann 2005a).

SE/SVY

`svy`[`(`[*vcetype*] [`,` *svy_options*]`)`] executes `oaxaca` while accounting for the survey settings identified by `svyset` (this is essentially equivalent to applying the `svy` prefix command, although the `svy` prefix is not allowed with `oaxaca` because of some technical issues). *vcetype* and *svy_options* are as described in [SVY] **svy**.

`vce(`*vcetype*`)` specifies the type of standard errors reported. *vcetype* can be `analytic` (the default), `robust`, `cluster` *clustvar*, `bootstrap`, or `jackknife`; see [R] ***vce_option***.

`cluster(`*varname*`)` adjusts standard errors for intragroup correlation; this is Stata 9 syntax for `vce(cluster` *clustvar*`)`.

`fixed`[`(`*varlist*`)`] identifies fixed regressors (all if specified without argument; an example for fixed regressors is experimental factors). The default is to treat regressors as stochastic. Stochastic regressors inflate the standard errors of the decomposition components.

[`no`]`suest` prevents or enforces using `suest` to obtain the covariances between the models or groups. `suest` is implied by `pooled`, `omega`, `reference()`, `svy`, `vce(cluster` *clustvar*`)`, and `cluster()`. Specifying `nosuest` can cause biased standard errors and is strongly discouraged.

`nose` suppresses the computation of standard errors.

Model estimation

`model1(`*model_opts*`)` and `model2(`*model_opts*`)` specify the estimation details for the two group-specific models. The syntax for *model_opts* is

[*estcom*] [, `addrhs(`*spec*`)` *estcom_options*]

where *estcom* is the estimation command to be used and *estcom_options* are options allowed by *estcom*. The default estimation command is `regress`. `addrhs(`*spec*`)` adds *spec* to the right-hand side of the model. For example, use `addrhs()` to add extra variables to the model. Here are some examples:

`model1(heckman, select(`*varlist_s*`) twostep)`

`model1(ivregress 2sls, addrhs((`*varlist2*`=`*varlist_iv*`)))`

`oaxaca` uses the first equation for the decomposition if a model contains multiple equations.

Furthermore, coefficients that occur in one of the groups are assumed to be zero for the other group. It is important, however, that the associated variables contain nonmissing values for all observations in both groups.

`noisily` displays the models' estimation output.

Reporting

`xb` displays a table containing the regression coefficients and predictor values on which the decomposition is based.

`level(`#`)` specifies the confidence level, as a percentage, for confidence intervals. The default is `level(95)` or as set by `set level`.

`eform` specifies that the results be displayed in exponentiated form.

`nolegend` suppresses the legend for the regressor sets defined by the `detail()` option.

(*Continued on next page*)

3.3 Saved results

Scalars

`e(N)`	number of observations	`e(N_1)`	number of obs. in group 1
`e(N_clust)`	number of clusters	`e(N_2)`	number of obs. in group 2

Macros

`e(cmd)`	`oaxaca`	`e(legend)`	definitions of regressor sets
`e(depvar)`	name of dependent variable	`e(adjust)`	names of adjustment variables
`e(by)`	name of group variable	`e(fixed)`	names of fixed variables
`e(group_1)`	value defining group 1	`e(suest)`	`suest`, if `suest` was used
`e(group_2)`	value defining group 2	`e(wtype)`	weight type
`e(title)`	title in estimation output	`e(wexp)`	weight expression
`e(model)`	type of decomposition	`e(clustvar)`	name of cluster variable
`e(weights)`	weights specified in `weight()`	`e(vce)`	*vcetype* specified in `vce()`
`e(refcoefs)`	equation name used in `e(b0)` for the reference coefficients	`e(vcetype)`	title used to label Std. Err.
		`e(properties)`	`b V`
`e(detail)`	`detail`, if detailed results were requested		

Matrices

`e(b)`	decomposition results	`e(b0)`	coefficients and X-values
`e(V)`	variance matrix of `e(b)`	`e(V0)`	variance matrix of `e(b0)`

Functions

`e(sample)`	marks estimation sample

4 Examples

Threefold decomposition

The standard application of the Blinder–Oaxaca technique is to divide the wage gap between, say, men and women into a part that is explained by differences in determinants of wages, such as education or work experience, and a part that cannot be explained by such group differences. An example using data from the Swiss Labor Market Survey 1998 (Jann 2003) is as follows:

```
. use oaxaca, clear
(Excerpt from the Swiss Labor Market Survey 1998)
. oaxaca lnwage educ exper tenure, by(female) noisily
Model for group 1
      Source |       SS       df       MS              Number of obs =     751
-------------+------------------------------           F(  3,   747) =  101.14
       Model |  49.613308     3  16.5377693           Prob > F      =  0.0000
    Residual | 122.143834   747  .163512495           R-squared     =  0.2889
-------------+------------------------------           Adj R-squared =  0.2860
       Total | 171.757142   750  .229009522           Root MSE      =  .40437

------------------------------------------------------------------------------
      lnwage |      Coef.   Std. Err.      t    P>|t|     [95% Conf. Interval]
-------------+----------------------------------------------------------------
        educ |   .0820549   .0060851    13.48   0.000      .070109    .0940008
       exper |   .0098347   .0016665     5.90   0.000     .0065632    .0131062
      tenure |   .0100314   .0020397     4.92   0.000     .0060272    .0140356
       _cons |    2.24205   .0778703    28.79   0.000      2.08918    2.394921
------------------------------------------------------------------------------
```

```
Model for group 2

      Source |       SS       df       MS              Number of obs =     683
-------------+------------------------------           F(  3,   679) =   40.34
       Model |  33.5197344     3  11.1732448           Prob > F      =  0.0000
    Residual |   188.08041   679  .276996185           R-squared     =  0.1513
-------------+------------------------------           Adj R-squared =  0.1475
       Total |  221.600144   682  .324926897           Root MSE      =   .5263

------------------------------------------------------------------------------
      lnwage |      Coef.   Std. Err.      t    P>|t|     [95% Conf. Interval]
-------------+----------------------------------------------------------------
        educ |   .0877579   .0087108    10.07   0.000     .0706546    .1048611
       exper |   .0131074   .0028971     4.52   0.000     .0074191    .0187958
      tenure |   .0036577   .0035374     1.03   0.301    -.0032878    .0106032
       _cons |   2.097806   .1091691    19.22   0.000     1.883457    2.312156
------------------------------------------------------------------------------

Blinder-Oaxaca decomposition                      Number of obs   =       1434
          1: female = 0
          2: female = 1

------------------------------------------------------------------------------
      lnwage |      Coef.   Std. Err.      z    P>|z|     [95% Conf. Interval]
-------------+----------------------------------------------------------------
Differential |
Prediction_1 |   3.440222   .0174874   196.73   0.000     3.405947    3.474497
Prediction_2 |   3.266761   .0218522   149.49   0.000     3.223932    3.309591
  Difference |   .1734607    .027988     6.20   0.000     .1186052    .2283163
-------------+----------------------------------------------------------------
Decomposit~n |
  Endowments |   .0852798    .015693     5.43   0.000     .0545222    .1160375
Coefficients |    .082563   .0255804     3.23   0.001     .0324263    .1326996
 Interaction |    .005618    .010966     0.51   0.608    -.0158749    .0271109
------------------------------------------------------------------------------
```

As is evident from the example, `oaxaca` first estimates two group-specific regression models and then performs the decomposition (the `noisily` option causes the group models' results to be displayed and is specified in the example for illustration). The default decomposition performed by `oaxaca` is the threefold decomposition (2). To compute the reverse threefold decomposition (3), specify `threefold(reverse)`.

The decomposition output reports the mean predictions by groups and their difference in the first panel. In our sample, the mean of log wages (`lnwage`) is 3.44 for men and 3.27 for women, yielding a wage gap of 0.17. In the second panel of the decomposition output, the wage gap is divided into three parts. The first part reflects the mean increase in women's wages if they had the same characteristics as men. The increase of 0.085 in the example indicates that differences in years of education (`educ`), work experience (`exper`), and job tenure (`tenure`) account for about half the wage gap. The second term quantifies the change in women's wages when applying the men's coefficients to the women's characteristics. The third part is the interaction term that measures the simultaneous effect of differences in endowments and coefficients.

Twofold decomposition

Alternatively, the twofold decomposition (4) can be requested, where `weight()`, `pooled`, or `omega` determines the choice of the reference coefficients. For example, `weight(1)` corresponds to (5), and `weight(0)` corresponds to (6). `omega` causes the coefficients from a pooled model over both samples to be used as the reference coefficients, which is equivalent to Oaxaca and Ransom's approach based on (7). The `pooled` option also causes the coefficients from a pooled model to be used, but now the pooled model also contains a group membership indicator. Based on the argumentation outlined in section 2, my suggestion is to use `pooled` rather than `omega`.

For our example data, the results after using the `pooled` option are as follows:

```
. oaxaca lnwage educ exper tenure, by(female) pooled
Blinder-Oaxaca decomposition                      Number of obs   =       1434

           1: female = 0
           2: female = 1

------------------------------------------------------------------------------
             |               Robust
      lnwage |      Coef.   Std. Err.      z    P>|z|     [95% Conf. Interval]
-------------+----------------------------------------------------------------
Differential |
Prediction_1 |   3.440222   .0174586   197.05   0.000     3.406004     3.47444
Prediction_2 |   3.266761   .0218042   149.82   0.000     3.224026    3.309497
  Difference |   .1734607   .0279325     6.21   0.000      .118714    .2282075
-------------+----------------------------------------------------------------
Decomposit~n |
   Explained |    .089347   .0137531     6.50   0.000     .0623915    .1163026
 Unexplained |   .0841137    .025333     3.32   0.001      .034462    .1337654
------------------------------------------------------------------------------
```

Again the conclusion is that differences in endowments account for about half the wage gap.[11]

A further possibility is to provide a stored reference model by using the `reference()` option. For example, for the decomposition of the wage gap between blacks and whites, the reference model is sometimes estimated based on all races, not just blacks and whites. Then the reference model would have to be estimated first using all observations and then be provided to `oaxaca` via the `reference()` option.

Exponentiated results

The results in the example above are expressed on the logarithmic scale (remember that log wages are used as the dependent variable), and it might be sensible to retransform the results to the original scale (here Swiss francs) by using the `eform` option:

11. Unlike the first example, robust standard errors are reported (`oaxaca` uses `suest` to estimate the joint variance matrix for all coefficients if `pooled` is specified; `suest` implies robust standard errors). To compute robust standard errors in the first example, you would have to add `vce(robust)` to the command.

```
. oaxaca, eform
Blinder-Oaxaca decomposition                    Number of obs   =       1434
          1: female = 0
          2: female = 1

------------------------------------------------------------------------------
             |               Robust
      lnwage |     exp(b)   Std. Err.      z    P>|z|     [95% Conf. Interval]
-------------+----------------------------------------------------------------
Differential |
Prediction_1 |   31.19388   .5446007   197.05   0.000     30.14454    32.27975
Prediction_2 |   26.22626   .5718438   149.82   0.000     25.12908    27.37135
  Difference |   1.189414   .0332234     6.21   0.000     1.126048    1.256346
-------------+----------------------------------------------------------------
Decomposit~n |
   Explained |    1.09346   .0150385     6.50   0.000     1.064379    1.123336
 Unexplained |   1.087753    .027556     3.32   0.001     1.035063    1.143125
------------------------------------------------------------------------------
```

The (geometric) means of wages are 31.2 Swiss francs for men and 26.2 Swiss francs for women, which amounts to a difference of 18.9%. Adjusting women's endowments levels to the levels of men would increase women's wages by 9.3%. A gap of 8.8% remains unexplained.

Survey estimation

`oaxaca` supports complex survey estimation, but `svy` has to be specified as an option and is not allowed as a prefix command (which does not restrict functionality). For example, the `wt` variable provides sampling weights for the Swiss Labor Market Survey 1998. The weights (and strata or primary sampling units [PSUs], if there were any) can be taken into account as follows:

(*Continued on next page*)

```
. svyset [pw=wt]

      pweight: wt
          VCE: linearized
  Single unit: missing
     Strata 1: <one>
         SU 1: <observations>
        FPC 1: <zero>

. oaxaca lnwage educ exper tenure, by(female) pooled svy

Blinder-Oaxaca decomposition

Number of strata   =         1                Number of obs      =      1647
Number of PSUs     =      1647                Population size    = 1657.1804
                                              Design df          =      1646

          1: female = 0
          2: female = 1

------------------------------------------------------------------------------
             |             Linearized
      lnwage |      Coef.   Std. Err.      t    P>|t|     [95% Conf. Interval]
-------------+----------------------------------------------------------------
Differential |
Prediction_1 |   3.405696   .0226311   150.49   0.000     3.361307    3.450085
Prediction_2 |   3.193847   .0276463   115.53   0.000     3.139622    3.248073
  Difference |   .2118488    .035728     5.93   0.000     .1417718    .2819259
-------------+----------------------------------------------------------------
Decomposit~n |
   Explained |   .1107614   .0189967     5.83   0.000     .0735011    .1480216
 Unexplained |   .1010875   .0315911     3.20   0.001     .0391246    .1630504
------------------------------------------------------------------------------
```

Detailed decomposition

Use the `detail` option to compute the individual contributions of the predictors to the components of the decomposition. `detail` specified without argument reports the contribution of each predictor individually. Alternatively, one can define groups of predictors for which the results can be subsumed in parentheses. Furthermore, one might apply the deviation contrast transform to dummy-variable sets so that the contribution of a categorical predictor to the unexplained part of the decomposition does not depend on the choice of the base category. For example,

```
. tabulate isco, nofreq generate(isco)
. oaxaca lnwage educ exper tenure isco2-isco9, by(female) pooled
> detail(exp_ten: exper tenure, isco: isco?) categorical(isco?)
Blinder-Oaxaca decomposition                     Number of obs   =       1434
           1: female = 0
           2: female = 1
```

lnwage	Coef.	Robust Std. Err.	z	P>|z|	[95% Conf.	Interval]
Differential						
Prediction_1	3.440222	.0174589	197.05	0.000	3.406003	3.474441
Prediction_2	3.266761	.0218047	149.82	0.000	3.224025	3.309498
Difference	.1734607	.0279331	6.21	0.000	.118713	.2282085
Explained						
educ	.0395615	.0097334	4.06	0.000	.0204843	.0586387
exp_ten	.0399316	.0089081	4.48	0.000	.022472	.0573911
isco	-.0056093	.012445	-0.45	0.652	-.0300009	.0187824
Total	.0738838	.017772	4.16	0.000	.0390513	.1087163
Unexplained						
educ	-.1324971	.1788045	-0.74	0.459	-.4829475	.2179533
exp_ten	.0129955	.0400811	0.32	0.746	-.0655619	.0915529
isco	-.0159367	.0296549	-0.54	0.591	-.0740592	.0421858
_cons	.2350152	.195018	1.21	0.228	-.1472132	.6172435
Total	.0995769	.0266887	3.73	0.000	.047268	.1518859

```
exp_ten: exper tenure
isco: isco1 isco2 isco3 isco4 isco5 isco6 isco7 isco8 isco9
```

Differences in education and combined differences in experience and tenure each account for about half the explained part of the outcome differential, whereas occupational segregation based on the nine major groups of the International Standard Classification of Occupations (ISCO-88) does not seem to matter much.

Selectivity bias adjustment

In labor-market research, it is common to include a correction for sample-selection bias in the wage equations based on the procedure by Heckman (1976, 1979). Wages are observed only for people who are participating in the labor force, and this might be a selective group. The most straightforward approach to account for selection bias in the decomposition is to deduct the selection effects from the overall differential and then apply the standard decomposition formulas to this adjusted differential (Reimers [1983]; an alternative approach is followed by Dolton and Makepeace [1986]; see Neuman and Oaxaca [2004] for an in-depth treatment of this issue).

(*Continued on next page*)

If `oaxaca` is used with `heckman`, the decomposition is automatically adjusted for selection. For example, the following command includes a selection correction in the wage equation for women and decomposes the adjusted wage gap. Labor-force participation (`lfp`) is modeled as a function of age, age squared, marital status, and the number of children at ages 6 or below and at ages 7 to 14.

```
. oaxaca lnwage educ exper tenure, by(female) model2(heckman, twostep
> select(lfp = age agesq married divorced kids6 kids714))
Blinder-Oaxaca decomposition                     Number of obs   =       1434
           1: female = 0
           2: female = 1
```

lnwage	Coef.	Std. Err.	z	P>|z|	[95% Conf.	Interval]
Differential						
Prediction_1	3.440222	.0174874	196.73	0.000	3.405947	3.474497
Prediction_2	3.275643	.0281554	116.34	0.000	3.220459	3.330827
Difference	.164579	.0331442	4.97	0.000	.0996176	.2295404
Decomposit~n						
Endowments	.0858436	.0157566	5.45	0.000	.0549613	.116726
Coefficients	.0736812	.031129	2.37	0.018	.0126695	.134693
Interaction	.0050542	.0109895	0.46	0.646	-.0164849	.0265932

Comparing the results with the output in the first example reveals that the uncorrected wages of women are slightly biased downward (3.267 versus the selectivity-corrected 3.276), and the wage gap is somewhat overestimated (0.173 versus the corrected 0.165).

It is sometimes sensible to compute the selection variables outside of `oaxaca` and then use the `adjust()` option to correct the differential (although here the selection variables are assumed known, which might slightly bias the standard errors). For example,

```
. probit lfp age agesq married divorced kids6 kids714 if female==1
  (output omitted)
. predict xb if e(sample), xb
(759 missing values generated)
. generate mills = normalden(-xb) / (1 - normal(-xb))
(759 missing values generated)
. replace mills = 0 if female==0
(759 real changes made)
. oaxaca lnwage educ exper tenure mills, by(female) adjust(mills)
Blinder-Oaxaca decomposition                    Number of obs    =      1434
                1: female = 0
                2: female = 1

------------------------------------------------------------------------------
      lnwage |      Coef.   Std. Err.      z    P>|z|     [95% Conf. Interval]
-------------+----------------------------------------------------------------
Differential |
Prediction_1 |   3.440222   .0174874   196.73   0.000     3.405947    3.474497
Prediction_2 |   3.266761   .0218659   149.40   0.000     3.223905    3.309618
  Difference |   .1734607   .0279987     6.20   0.000     .1185843    .2283372
    Adjusted |    .164579    .033215     4.95   0.000     .0994788    .2296792
-------------+----------------------------------------------------------------
Decomposit~n |
  Endowments |   .0858436   .0157766     5.44   0.000     .0549221    .1167651
Coefficients |   .0736812   .0312044     2.36   0.018     .0125217    .1348407
 Interaction |   .0050542   .0110181     0.46   0.646    -.0165409    .0266493
------------------------------------------------------------------------------
```

Using oaxaca with nonstandard models

You can also use `oaxaca`, for example, with binary outcome variables and employ a command such as `logit` to estimate the models. You have to understand, however, that `oaxaca` will always apply the decomposition to the linear predictions from the models (based on the first equation if a model contains multiple equations). With `logit` models, for example, the decomposition computed by `oaxaca` is expressed in terms of log odds and not in terms of probabilities or proportions. Approaches to decompose differences in proportions are provided by, e.g., Gomulka and Stern (1990), Fairlie (2005), or Yun (2005a). Also see Sinning, Hahn, and Bauer (in this issue) if you are interested in decomposing group differences in categorical or limited outcome variables.

For binary outcomes, as an anonymous reviewer of this article pointed out, a convenient alternative approach might be to use `oaxaca` with the linear probability model. Here the decomposition results are on the probability scale (see, e.g., Long [1997, 35–40] or Wooldridge [2003, 240–245] on the pros and cons of the linear probability model).

(*Continued on next page*)

5 Acknowledgments

I would like to thank Debra Hevenstone and Austin Nichols for their comments and suggestions.

6 References

Blinder, A. S. 1973. Wage discimination: Reduced form and structural estimates. *Journal of Human Resources* 8: 436–455.

Cotton, J. 1988. On the decomposition of wage differentials. *Review of Economics and Statistics* 70: 236–243.

Daymont, T. N., and P. J. Andrisani. 1984. Job preferences, college major, and the gender gap in earnings. *Journal of Human Resources* 19: 408–428.

Dolton, P. J., and G. H. Makepeace. 1986. Sample selection and male–female earnings differentials in the graduate labour market. *Oxford Economic Papers* 38: 317–341.

Fairlie, R. W. 2005. An extension of the Blinder–Oaxaca decomposition technique to logit and probit models. *Journal of Economic and Social Measurement* 30: 305–316.

Fortin, N. M. 2006. Greed, altruism, and the gender wage gap. http://www.econ.ubc.ca/nfortin/Fortinat8.pdf.

Gardeazabal, J., and A. Ugidos. 2004. More on identification in detailed wage decompositions. *Review of Economics and Statistics* 86: 1034–1036.

Gomulka, J., and N. Stern. 1990. The employment of married women in the United Kingdom, 1970–1983. *Econometrica* 57: 171–199.

Greene, W. H. 2008. *Econometric Analysis*. 6th ed. Upper Saddle River, NJ: Prentice Hall.

Gujarati, D. N. 2003. *Basic Econometrics*. 3rd ed. New York: McGraw–Hill.

Haisken-DeNew, J. P., and C. M. Schmidt. 1997. Interindustry and interregion differentials: Mechanics and interpretation. *Review of Economics and Statistics* 79: 516–521.

Hardy, M. A. 1993. *Regression With Dummy Variables*. Newbury Park, CA: Sage.

Heckman, J. J. 1976. The common structure of statistical models of truncation, sample selection and limited dependent variables and a simple estimator for such models. *Annals of Econometrics and Social Measurement* 5: 475–492.

———. 1979. Sample selection bias as a specification error. *Econometrica* 47: 153–161.

Heinrichs, J., and P. Kennedy. 2007. A computational trick for calculating the Blinder–Oaxaca decomposition and its standard error. *Economics Bulletin* 3(66): 1–7.

Hendrickx, J. 1999. dm73: Using categorical variables in Stata. *Stata Technical Bulletin* 52: 2–8. Reprinted in *Stata Technical Bulletin Reprints*, vol. 9, pp. 51–59. College Station, TX: Stata Press.

Horrace, W. C., and R. L. Oaxaca. 2001. Inter-industry wage differentials and the gender wage gap: An identification problem. *Industrial and Labor Relations Review* 54: 611–618.

Jackson, J. D., and J. T. Lindley. 1989. Measuring the extent of wage discrimination: A statistical test and a caveat. *Applied Economics* 21: 515–540.

Jann, B. 2003. The Swiss labor market survey 1998 (SLMS 98). *Journal of Applied Social Science Studies* 123: 329–335.

———. 2005a. devcon: Stata module to apply the deviation contrast transform to estimation results. Boston College Department of Economics, Statistical Software Components S450603. Downloadable from http://ideas.repec.org/c/boc/bocode/s450603.html.

———. 2005b. Standard errors for the Blinder–Oaxaca decomposition. 2005 German Stata Users Group meeting. http://repec.org/dsug2005/oaxaca_se_handout.pdf.

Jones, F. L. 1983. On decomposing the wage gap: A critical comment on Blinder's method. *Journal of Human Resources* 18: 126–130.

Jones, F. L., and J. Kelley. 1984. Decomposing differences between groups: A cautionary note on measuring discrimination. *Sociological Methods and Research* 12: 323–343.

Kennedy, P. 1986. Interpreting dummy variables. *Review of Economics and Statistics* 68: 174–175.

Lin, E. S. 2007. On the standard errors of Oaxaca-type decompositions for inter-industry gender wage differentials. *Economics Bulletin* 10(6): 1–11.

Long, J. S. 1997. *Regression Models for Categorical and Limited Dependent Variables*. Thousand Oaks, CA: Sage.

Mood, A. M., F. A. Graybill, and D. C. Boes. 1974. *Introduction to the Theory of Statistics*. 3rd ed. New York: McGraw–Hill.

Neuman, S., and R. L. Oaxaca. 2004. Wage decompositions with selectivity-corrected wage equations: A methodological note. *Journal of Economic Inequality* 2: 3–10.

Neumark, D. 1988. Employers' discriminatory behavior and the estimation of wage discrimination. *Journal of Human Resources* 23: 279–295.

Nielsen, H. S. 2000. Wage discrimination in Zambia: An extension of the Oaxaca–Blinder decomposition. *Applied Economics Letters* 7: 405–408.

Oaxaca, R. 1973. Male–female wage differentials in urban labor markets. *International Economic Review* 14: 693–709.

Oaxaca, R. L., and M. R. Ransom. 1994. On discrimination and the decomposition of wage differentials. *Journal of Econometrics* 61: 5–21.

———. 1998. Calculation of approximate variances for wage decomposition differentials. *Journal of Economic and Social Measurement* 24: 55–61.

———. 1999. Identification in detailed wage decompositions. *Review of Economics and Statistics* 81: 154–157.

O'Donnell, O., E. van Doorslaer, A. Wagstaff, and M. Lindelow. 2008. *Analyzing Health Equity Using Household Survey Data: A Guide to Techniques and Their Implementation*. Washington, DC: The World Bank.

Polavieja, J. G. 2005. Task specificity and the gender wage gap: Theoretical considerations and empirical analysis of the Spanish survey on wage structure. *European Sociological Review* 21: 165–181.

Reimers, C. W. 1983. Labor market discrimination against Hispanic and black men. *Review of Economics and Statistics* 65: 570–579.

Shrestha, K., and C. Sakellariou. 1996. Wage discrimination: A statistical test. *Applied Economics Letters* 3: 649–651.

Silber, J., and M. Weber. 1999. Labour market discrimination: Are there significant differences between the various decomposition procedures? *Applied Economics* 31: 359–365.

Sinning, M., M. Hahn, and T. K. Bauer. 2008. The Blinder–Oaxaca decomposition for nonlinear regression models. *Stata Journal* 8: 480–492.

Stanley, T. D., and S. B. Jarrell. 1998. Gender wage discrimination bias? A meta-regression analysis. *Journal of Human Resources* 33: 947–973.

Suits, D. B. 1984. Dummy variables: Mechanics v. interpretation. *Review of Economics and Statistics* 66: 177–180.

Weesie, J. 1999. sg121: Seemingly unrelated estimation and the cluster-adjusted sandwich estimator *Stata Technical Bulletin* 52: 34–47. Reprinted in *Stata Technical Bulletin Reprints*, vol. 9, pp. 231–248. College Station, TX: Stata Press.

Weichselbaumer, D., and R. Winter-Ebmer. 2005. A meta-analysis of the international gender wage gap. *Journal of Economic Surveys* 19: 479–511.

Winsborough, H. H., and P. Dickenson. 1971. Components of negro–white income differences. In *Proceedings of the Social Statistics Section*, 6–8. Washington, DC: American Statistical Association.

Wooldridge, J. M. 2003. *Introductory Econometrics: A Modern Approach*. 2nd ed. New York: Thomson Learning.

Yun, M.-S. 2005a. Hypothesis tests when decomposing differences in the first moment. *Journal of Economic and Social Measurement* 30: 295–304.

———. 2005b. A simple solution to the identification problem in detailed wage decompositions. *Economic Inquiry* 43: 766–772.

About the author

Ben Jann is a sociologist at ETH Zürich in Zürich, Switzerland.
